# NELF and PAF1C complexes are core transcriptional machineries controlling colon cancer stemness

**DOI:** 10.1038/s41388-023-02930-0

**Published:** 2024-01-05

**Authors:** Koji Aoki, Akari Nitta, Ayumi Igarashi

**Affiliations:** Department of Pharmacology, Faculty of Medicine, University of Fukui, Fukui, Japan

**Keywords:** Cancer stem cells, Mechanisms of disease

## Abstract

Mutations in *APC*, found in 80% of colon caner, enhance β-catenin stabilization, which is the initial step of colonic tumorigenesis. However, the core transcriptional mechanism underlying the induction of colon cancer stemness by stable β-catenin remains unclear. Here, we found that inducible inhibition of β-catenin suppressed elongation of Pol II and RNA polymerase-associated factor 1 complex (PAF1C) around the transcription start site (TSS) of *LGR5*. Moreover, stable β-catenin enhanced the formation of active Pol II complex cooperatively with CDC73 and CDK9 by facilitating the recruitment of DRB sensitivity-inducing factor (DSIF) and negative elongation factor (NELF) complexes to the Pol II complex. Subsequently, stable β-catenin facilitated the formation of the Pol II–DSIF–PAF1C complex, suggesting that stable β-catenin induces cancer stemness by stimulating active Pol II complex through NELF and PAF1C. Furthermore, NELF or PAF1C inhibition recapitulated the changes in cancer stemness-related gene expression induced by the inhibition of stable β-catenin and suppressed colon cancer stemness. Additionally, the chemical inhibition of CDK12 (a downstream transcription CDK of PAF1C) suppressed colon cancer stemness. These results suggest that NELF and PAF1C are the core transcriptional machineries that control expression of colon cancer stemness-inducing genes and may be therapeutic targets for colon cancer.

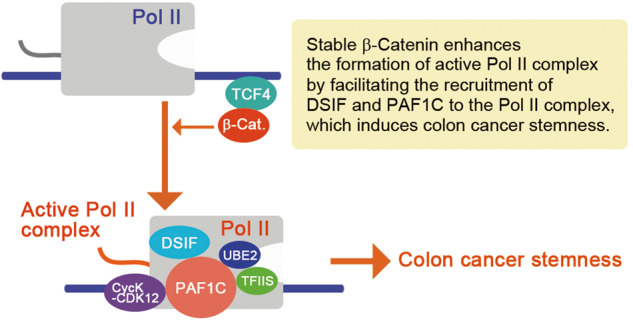

## Introduction

Colorectal cancer (CRC) cases are classified as hypermutated (16% of all sporadic CRC cases) or non-hypermutated (84% of all sporadic CRC cases) [1]. Mutations in the *APC* gene have been reported in approximately 80% of all sporadic non-hypermutated CRC, 50% of sporadic hypermutated CRC, and familial adenomatous polyposis (FAP) cases [1]. Its protein product APC acts as a scaffold of the destruction complex that promotes GSK3β-mediated phosphorylation and ubiquitin-dependent degradation of β-catenin (encoded by *CTNNB1*) [2]. Mutations in *APC* or *CTNNB1* enhance the stabilization of β-catenin and its nuclear accumulation. This in turn induces the expression of genes that increase cancer cell stemness, resulting in the initiation of colonic tumorigenesis [3–5]. Nuclear β-catenin-induced gene expression requires β-catenin to interact with TCF transcription factors because β-catenin does not contain a DNA-binding domain [3–5]. However, inhibition of nuclear β-catenin by dominant-negative TCF has not been recapitulated by inhibiting other transcription-related factors, and the core transcriptional mechanism underlying the control of cancer stemness by nuclear β-catenin remains unclear.

The mRNA levels are determined by transcription activation, initiation complex assembly, and productive elongation of mRNA [6]. In genes such as those involved in developmental regulation, the RNA polymerase II (Pol II) is paused between initiation complex assembly and productive elongation at a 30–50 nucleotide region downstream of TSS [7]. Therefore, the release of Pol II is a critical step for the initiation of productive elongation [8]. Pol II pausing in the promoter proximal regions is regulated by various complexes such as DSIF, NELF, PAF1C, and CDK9–cyclin T, which is also known as positive elongation factor-b (pTEFb) [8, 9]. The DSIF complex consists of SPT4 and SPT5 [10], whereas the NELF complex consists of NELFA, NELFB, NELFCD, and NELFE [11]. PAF1C consists of PAF1, CTR9, CDC73, RTF1, LEO1, and WDR61 [12]. DSIF and NELF are required for Pol II pausing, whereas DSIF and PAF1C are required for Pol II release [8, 9]. PAF1C stimulates its self-recruitment to the DSIF–NELF–Pol II complex, leading to the association of Pol II complex with pTEFb and cyclin K–CDK12 [9]. PAF1C also contributes to the monoubiquitination of H2B at Lys120 (H2BK120ub) by the E2 ubiquitin-conjugating enzyme UBE2 via an interaction with the E3 ubiquitin-protein ligases RNF20 and RNF40 [8]. In this study, we investigated the transcriptional mechanism underlying the control of colon cancer stemness by stable β-catenin through the NELF and PAF1 complexes.

## Results

### Decreased occupancy levels of Pol II and PAF1 on *LGR5* upon *CTNNB1* knockdown

**Fig. 1:**
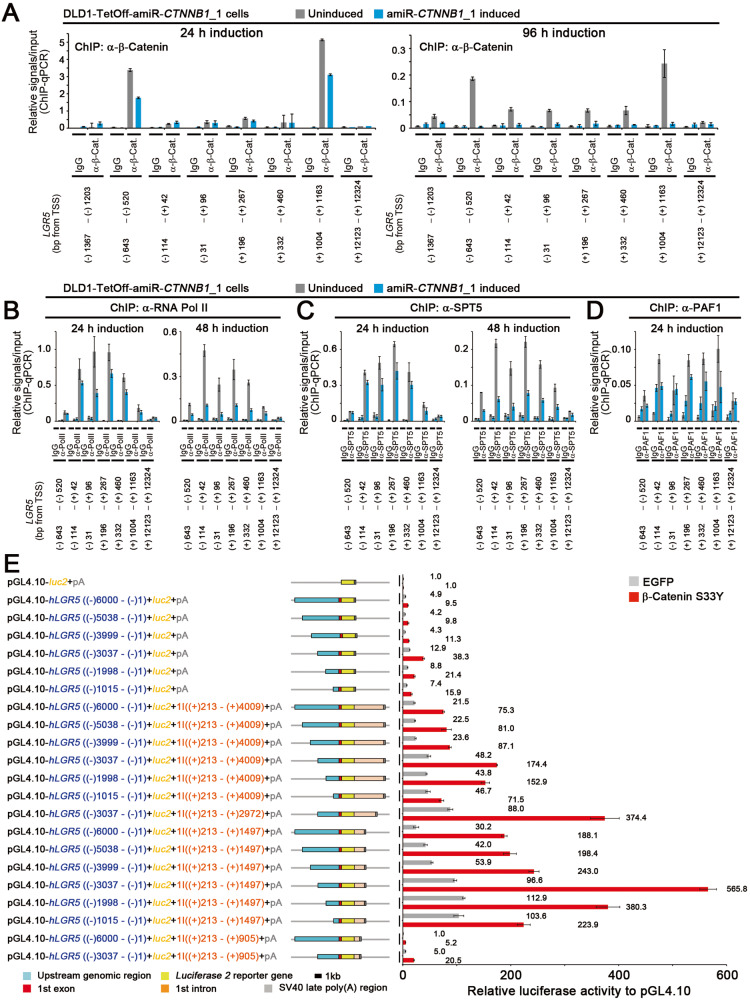
Decreased occupancy levels of Pol II and PAF1 on *LGR5* upon *CTNNB1* knockdown. **A** Bar graphs of the ChIP-qPCR data showing the relative occupancy level of β-catenin (mean ± SD) on the indicated positions of the *LGR5* gene upon expression of amiR-*CTNNB1*_1 (blue) in DLD1-TetOff cells for 24 (left) and 96 h (right), compared with that in cells not expressing the amiRNA (gray). IgG was used as the negative control. Bar graphs of the ChIP-qPCR data showing the relative occupancy levels (mean ± SD) of Pol II (**B**), SPT5 (**C**), and PAF1 (**D**) on the indicated positions of *LGR5* upon expression of amiR-*CTNNB1*_1 (blue) in DLD1-TetOff cells for the indicated hours, compared with those in cells not expressing the amiRNA (gray). IgG was used as the negative control. **E** The *LGR5 luc* reporters and a bar graph showing the luciferase activity (mean ± SD) of *LGR5 luc* reporters relative to that of pGL4.10-*luc2* control upon expression of β-catenin-S33Y in 293 T cells. Luciferase activities were determined at 40 h after the *luc* reporter transfection.

To understand the transcriptional mechanism underlying the induction of colon cancer stemness, we constructed TetOff cells that conditionally expressed an artificially-designed microRNA (amiRNA or amiR) targeting the open reading frame of *CTNNB1* (Fig. 1A). Additionally, we analyzed the binding dynamics of Pol II on the *LGR5* gene upon inducible knockdown of *CTNNB1* (Fig. 1B). We found that upon *CTNNB1* knockdown for 24 h, Pol II occupancy level decreased strongly at position −31 to +96 of *LGR5* and decreased mildly at position −114 to +42 (Fig. 1B). Upon *CTNNB1* knockdown for 48 h, it decreased further. The occupancy level of SPT5 showed similar dynamics (Fig. 1C). Likewise, the *CTNNB1* knockdown also decreased PAF1 occupancy level on the *LGR5* gene (Fig. 1D), suggesting that *CTNNB1* knockdown suppressed Pol II elongation from the promoter proximal region in *LGR5* possibly via the PAF1C inhibition. To analyze the underlying mechanism, we constructed various *LGR5 luciferase (luc)* reporters (Fig. 1E) by sandwiching *luc* between the highly β-catenin-occupied regions found approximately 500 base pairs (bp) upstream and 1000 bp downstream of the *LGR5* TSS (Fig. 1A). We found that β-catenin mutant carrying a substitution of tyrosine for serine at codon 33 (β-catenin-S33Y) increased the activities of the *LGR5 luc* reporters (Fig. 1E). β-Catenin-S33Y is more stable than its wild type because GSK3β phosphorylates some serine and threonine residues at the n-terminal region of β-catenin including codon 33, which induces ubiquitination and proteasome-mediated degradation of β-catenin [2, 13].

### Control of colon cancer stemness by PAF1

**Fig. 2:**
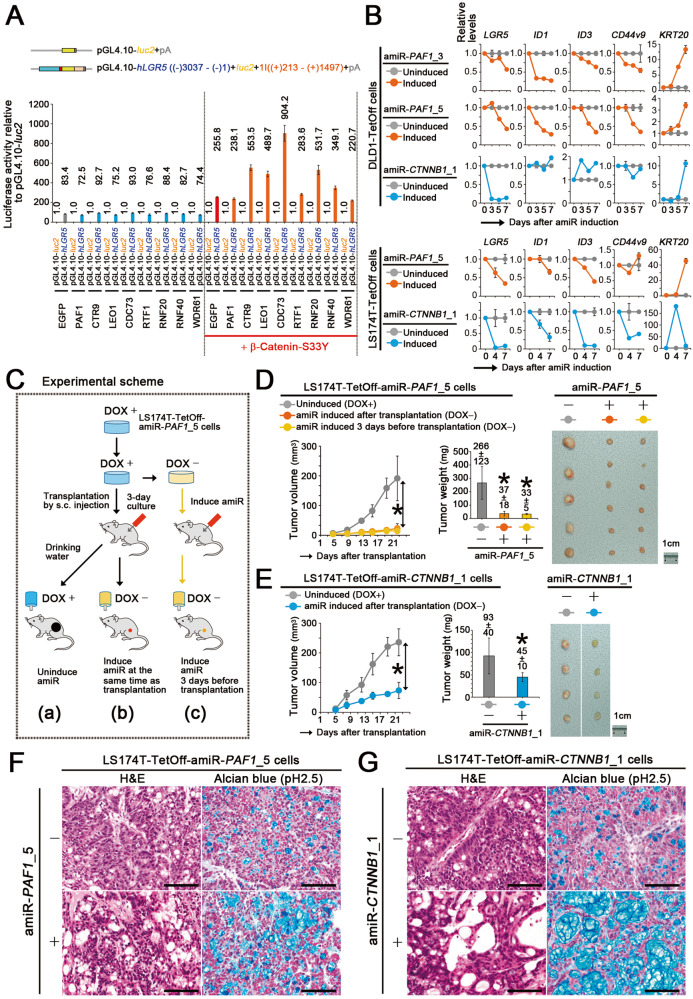
Control of colon cancer stemness by PAF1. **A** Bar graph showing the luciferase activity (mean ± SD) of *LGR5 luc* reporter relative to that of pGL4.10-*luc2* control upon expression of PAF1C components or its related factors with co-expression of β-catenin-S33Y in 293 T cells. The red line at the bottom indicates the additional expression of β-catenin-S33Y. Luciferase activity was determined at 40 h after the *luc* reporter transfection. **B** Line graphs of the qPCR data showing the relative expression levels (mean ± SD) of cancer stemness-related or differentiation marker genes upon expression of the respective amiRNAs targeting *PAF1* (orange) or *CTNNB1* (blue) in DLD1-TetOff (top) and LS174T-TetOff (bottom) cells for the indicated days, compared with those in cells not expressing the amiRNAs (gray). **C** Illustration of the experimental procedure of the subcutaneous transplantation of LS174T-TetOff-amiR-*PAF1*_5 cells into immunodeficient mice. In (b), an amiRNA targeting *PAF1* was expressed at the same time as transplantation, whereas it was expressed 3 days before transplantation in (c), compared with uninduced cells in (a). Mean volume (left) and weight (center) of the subcutaneously transplanted tumors of LS174T-TetOff cells that expressed the amiRNAs targeting *PAF1* (**D**; orange and yellow) or *CTNNB1* (**E**; blue) compared with those in tumors not expressing the amiRNAs (gray). Data represent the mean ± SD from 6−8 tumors. Dissection micrographs (right) showing the excised LS174T-TetOff tumors at 22 days post-transplantation. Scale bars, 1 cm. *P < 0.05, compared with tumors not expressing amiRNAs. In **D**, **E**, orange and blue indicate the tumors with the expression of the amiRNAs after transplantation, whereas yellow indicates the tumors with the amiRNA expression 3 days before transplantation. Gray indicates the tumors without the expression of the amiRNAs. Optical micrographs showing the tissue sections of the transplanted tumors of LS174T-TetOff cells that expressed amiRNAs targeting *PAF1* (**F**; bottom) or *CTNNB1* (**G**; bottom) compared with those of tumors not expressing the amiRNAs (top). Hematoxylin and eosin (H&E, left) and alcian blue stain (pH 2.5, right). Scale bars, 200 μm.

We investigated the potential involvement of PAF1C in controlling *LGR5* expression. Notably, expression of CTR9, LEO1, CDC73, RNF20, and RNF40 increased *LGR5 luc* reporter activity upon their simultaneous expression with β-catenin-S33Y, whereas none of them showed this effect without β-catenin-S33Y expression (Fig. 2A). These results suggest that PAF1C is required for the induction of *LGR5* expression by stable β-catenin. To test this hypothesis, we constructed TetOff cells that conditionally expressed amiRNAs targeting the open reading frame of *PAF1* as PAF1 is one of the major scaffolding components within PAF1C [14, 15]. Inducible expression of the amiRNAs decreased the *PAF1* expression level to approximately 20% of that in uninduced cells and inhibited the proliferation of TetOff cells (Supplementary Fig. 1A, B). Suppression of PAF1C function was also confirmed by the reduction in H2BK120ub level (Supplementary Fig. 1C). Moreover, *PAF1* knockdown decreased the expression levels of the genes such as *LGR5*, *ID1*, *ID3*, *CD44v9*, *CD133*, *BMI1*, *RNF43*, *EPHB2*, *SOX9*, and *ASCL2* (Fig. 2B and Supplementary Fig. 2A), which are critical for inducing colon cancer stemness and its markers [5, 16]. In contrast, it increased the expression levels of intestinal epithelial differentiation markers such as *KRT20* and *MUCs*. These changes in gene expression essentially recapitulated those observed upon the knockdown of *CTNNB1* (Fig. 2B and Supplementary Fig. 2A, B), suggesting that PAF1C plays a key role in controlling the expression of genes that are essential for colon cancer stemness and is induced by stable β-catenin. *CTNNB1* knockdown in DLD1-TetOff cells did not decrease *ID1*, *ID3*, *PHLDA1*, and *BMI1* expression levels, whereas *PAF1* knockdown did (Fig. 2B and Supplementary Fig. 2A, B). In contrast, *PAF1* knockdown did not decrease the expression levels of *ZNFRF3* and *c-MYC* whereas *CTNNB1* knockdown did. These results suggest that PAF1C controls expression of some genes such as *ID1* and *ID3* independent of β-catenin and that PAF1C does not contribute to the expression of all the genes controlled by stable β-catenin.

To analyze the effects of suppressing PAF1C on in vivo tumor formation, we subcutaneously transplanted LS174T-TetOff-amiR-*PAF1*_5 cells into immunodeficient mice (Fig. 2C) as LS174T cells have higher transplantability than DLD1 cells (data not shown). Continuous knockdown of *PAF1* reduced tumor weight and size to less than 20% (Fig. 2D), which was a stronger effect than that observed for continuous knockdown of *CTNNB1* (Fig. 2E). Besides, knockdown of *PAF1* or *CTNNB1* increased the number of cells stained with alcian blue (Fig. 2F, G), which indicates the production of acid mucins by goblet cells in the intestines. This observation was consistent with the increase in *MUC* expression levels upon knockdown of *PAF1* or *CTNNB1* (Supplementary Fig. 2A). These results collectively suggest that PAF1 maintains the stemness of colon cancer cells.

### Control of colon cancer stemness by PAF1C and its downstream effectors

**Fig. 3:**
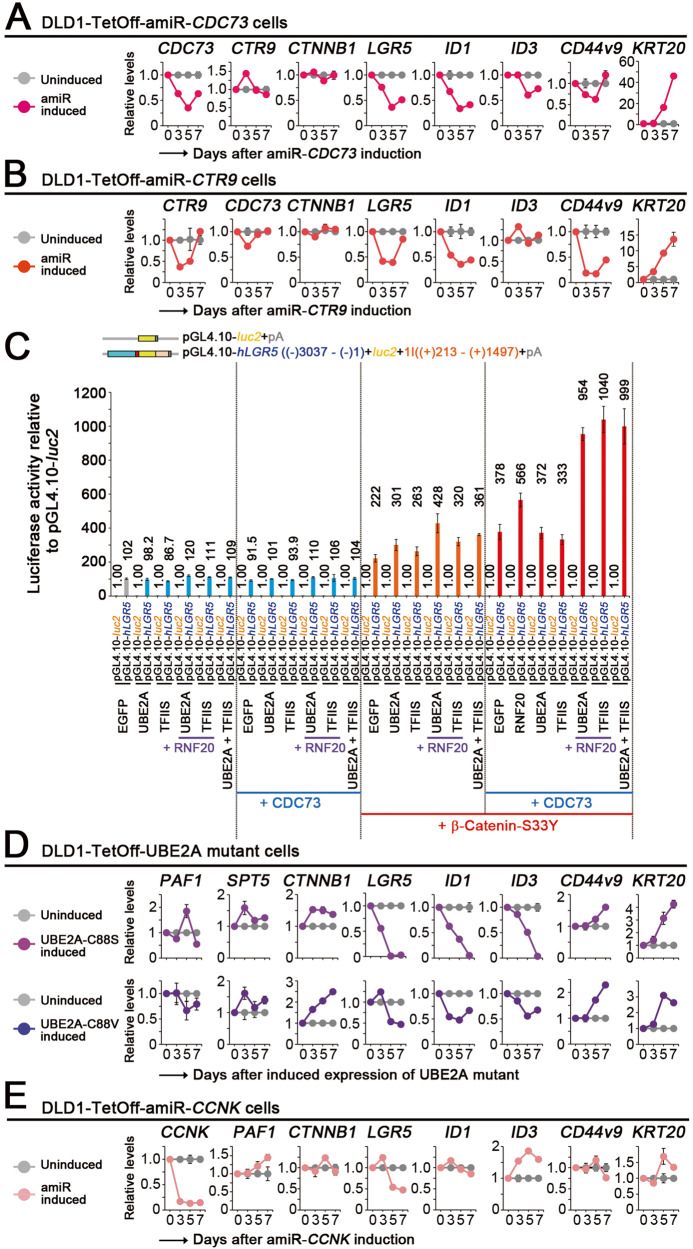
Control of colon cancer stemness by PAF1C and its downstream effectors. Line graphs of the qPCR data showing the relative expression levels (mean ± SD) of cancer stemness-related or differentiation marker genes upon expression of amiR-*CDC73* (**A**; red) or amiR-*CTR9* (**B**; orange) in DLD1-TetOff cells for the indicated days, compared with those in cells not expressing the amiRNAs (gray). **C** Bar graph showing the luciferase activity (mean ± SD) of the *LGR5 luc* reporter relative to that of pGL4.10-*luc2* control upon combinational expression of PAF1C components, UBE2A, TFIIS, and β-catenin-S33Y in 293 T cells. Red, blue, and purple lines indicate the additional expression of β-catenin-S33Y, CDC73, and RNF20, respectively. Luciferase activity was determined at 40 h after the *luc* reporter transfection. Line graphs of the qPCR data showing the relative expression levels (mean ± SD) of cancer stemness-related genes upon expression of UBE2A-C88S, UBE2A-C88V (**D**; purple), or an amiR-*CCNK* (**E**; pink) in DLD1-TetOff cells for the indicated days, compared with those in cells not expressing them (gray).

Among the PAF1C components, CDC73 is known to interact with β-catenin [17] and showed the strongest stimulation on the *LGR5 luc* reporter (Fig. 2A). CTR9 acts as a platform for PAF1C [6] and showed the strong stimulation on the *LGR5 luc* reporter as well (Fig. 2A). To determine the mechanism underlying colon cancer stemness control by PAF1C, we further generated TetOff cells conditionally expressing amiRNAs against *CDC73* and *CTR9* (Fig. 3A, B). Upon expression of the amiRNAs, the expression levels of *CDC73* and *CTR9* decreased on day 3 or 5 to approximately 35% of those in uninduced cells (Fig. 3A, B). In a similar manner to the *PAF1* knockdown, *CDC73* or *CTR9* knockdown inhibited cell proliferation, reduced the expression levels of most cancer stem cell marker genes including *LGR5* and *ID1*, and increased those of *KRT20* and *MUCs* (Fig. 3A, B and Supplementary Fig. 3A, B). These changes in gene expression essentially recapitulated those observed upon the knockdown of *PAF1*, whereas *CDC73* or *CTR9* knockdown also decreased *ZNFRF3* expression level. This effect may be attributed to stronger suppression of PAF1C function as a result of *CDC73* or *CTR9* knockdown than *PAF1* knockdown because expression of the former genes elicited a strong increase in *LGR5 luc* reporter activity (Fig. 2A).

The PAF1C downstream molecules RNF20, TFIIS, and UBE2A exhibited a cooperative interaction with stable β-catenin and CDC73 to elicit a strong and synergistic increase in *LGR5 luc* reporter activity (Fig. 3C). These results are consistent with an earlier report describing a cooperative interaction between PAF1C and TFIIS [14]. Next, we then generated DLD1-TetOff cells that conditionally expressed human UBE2A mutants carrying substitutions for cysteine at the active site at position 88 (C88S and C88V) [18]. Expression of the UBE2A mutants blocked H2B monoubiquitylation, which is the downstream histone modification of PAF1C [19], and inhibited the proliferation of the TetOff cells (Supplementary Fig. 4A, B). Crucially, expression of UBE2A mutants reduced the expression levels of most cancer stem cell marker genes including those of *LGR5* and *ID1* and increased those of *KRT20* and *MUCs* (Fig. 3D and Supplementary Fig. 4C). Moreover, we knocked down *CCNK* whose protein product cyclin K forms a cyclin K–CDK12 complex, which is a PAF1C-downstream transcriptional cyclin–CDK complex [9]. Knockdown of *CCNK* inhibited the proliferation of the TetOff cells, reduced the expression levels of *LGR5* and some stem cell markers such as *CD133* and *ALDH1B*, and increased those of *KRT20* and *MUCs* (Fig. 3E and Supplementary Fig. 4D, E). Collectively, these results suggest that PAF1C and its downstream effectors contribute to controlling the expression of genes that are critical for the induction of colon cancer stemness. However, expression of UBE2A mutants or knockdown of *CCNK* increased the expression levels of some stem cell marker genes, namely, *ID1*, *ID3*, *CD44v9*, and *BMI1*, suggesting that inhibiting one of the PAF1C downstream effectors is insufficient at recapitulating the effects of PAF1C inhibition.

### Enhanced formation of Pol lI–DSIF–PAF1C complex by stable β-catenin

**Fig. 4:**
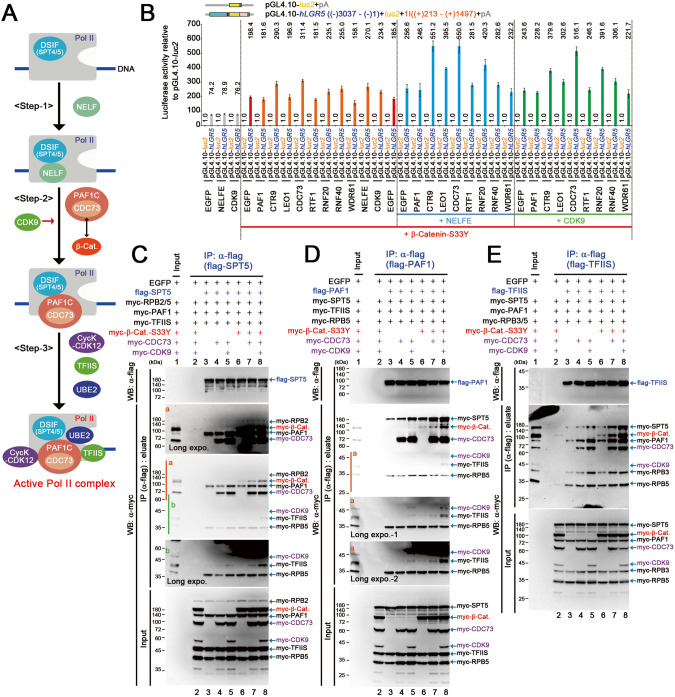
Enhanced formation of Pol II–DSIF–PAF1C complex by stable β-catenin. **A** Illustration of a current model of the formation of active Pol II complex containing PAF1C, DSIF, TFIIS, and UBE2. After the recruitment of the DSIF complex to Pol II, NELF binds to the Pol II–DSIF complex (Step-1). After the release of NELF from the Pol II complex, the Pol II–DSIF complex is bound with PAF1C (Step-2) to form a complex with TFIIS, UBE2, and cycin K (CycK)–CDK12 (Step-3). β-Catenin interacts with CDC73 [17]. **B** Bar graph showing the luciferase activity (mean ± SD) of *LGR5 luc* reporter relative to that of pGL4.10-*luc*2 control upon expression of PAF1C components, NELF, and CDK9 in 293 T cells. Red, blue, and green lines indicate the additional expression of β-catenin-S33Y, NELFE, and CDK9, respectively. Luciferase activity was determined at 40 h after the *luc* reporter transfection. Immunoblot images of the immunoprecipitation (IP) assays showing the effects of β-catenin-S33Y (β-Cat.), CDC73, and CDK9 on the complex formation of SPT5 (**C**) and PAF1 (**D**) containing TFIIS. The cells were harvested on day 2 after transfection of the plasmid vectors that expressed the indicated proteins into 293 T cells, and IP was performed. After IP of flag-SPT5 (**C**) or flag-PAF1 (**D**), the amounts of the co-immunoprecipitated myc-tagged molecules were analyzed via immunoblotting. Longer exposure gel images (a and b) were shown. **E** Immunoblot images of the IP assays showing the effects of β-catenin-S33Y (β-Cat.), CDC73, and CDK9 on the complex formation of TFIIS with SPT5 and PAF1. After IP of flag-TFIIS, the amounts of the co-immunoprecipitated myc-tagged molecules were analyzed via immunoblotting.

We further analyzed the mechanisms underlying the regulation of PAF1C function by stable β-catenin, by analyzing the formation of active Pol II complex (Fig. 4A) [6]. We developed an immunoprecipitation assay by expressing molecules that are essential for the active Pol II complex formation. We included the following proteins: SPT5 from DSIF; PAF1 and CDC73 from PAF1C; RPB2, 3, and 5 from PoI II subunits; and TFIIS and UBE2A, whose immunoblot signals could be discriminated (Supplementary Fig. 5A). As components of the active Pol II–DSIF–PAF1C complex, TFIIS and UBE2A play critical roles in transcription [14]; therefore, we assumed that the amounts of TFIIS and UBE2A bound with SPT5 or PAF1 correlated with the amount of active Pol II complex (Fig. 4A). Additionally, β-catenin-S33Y was expressed because it induced the PAF1C-mediated increase in *LGR5 luc* reporter activity (Fig. 2A).

In a preliminary experiment, we expressed flag-tagged SPT5 with myc-tagged PAF1, RPB5, TFIIS, and UBE2A. As with expression of PAF1, UBE2A, and RPB5, the amount of flag-SPT5-bound TFIIS was low (Supplementary Fig. 5B; lane 3); however, its amount increased by approximately three times upon expression of CDC73 (Supplementary Fig. 5B; lane 4) and by >10 times upon expression of β-catenin-S33Y (lanes 5 and 6). The level of flag-SPT5-bound UBE2A also increased upon expression of CDC73 or β-catenin-S33Y; however, it was too low to be quantified (Supplementary Fig. 5B; lanes 4–6). Accordingly, we used TFIIS as the indicator of the active Pol II complex formation. We also screened the effectors that control *LGR5* expression and identified CDK9 and NELFE as the activators of the *LGR5 luc* reporter (Fig. 4B).

We first expressed flag-SPT5 with myc-tagged PAF1, TFIIS, and RPBs. Expression of CDC73 and NELFE cooperatively with CDK9 increased the amounts of flag-SPT5-bound RPB2, PAF1, and TFIIS (Fig. 4C and Supplementary Fig. 5C; lanes 4 and 5), which was further enhanced by β-catenin-S33Y (lanes 7 and 8).

Next, we expressed flag-PAF1 with myc-tagged SPT5, TFIIS, and RPBs. As observed when expressing flag-SPT5, expression of CDC73 and NELFE cooperatively with CDK9 increased the amounts of flag-PAF1-bound RPB2, SPT5, and TFIIS (Fig. 4D and Supplementary Fig. 5D, E; lanes 4 and 5), which was enhanced further by β-catenin-S33Y (lanes 7 and 8). These results suggest that stable β-catenin promotes the formation of active Pol II complex via enhanced recruitment of DSIF and PAF1C to the Pol II complex cooperatively with CDC73, NELFE, and CDK9. To confirm this interpretation, we expressed flag-TFIIS with myc-tagged SPT5, PAF1, and RPBs and found that expression of CDC73 and NELFE cooperatively with CDK9 increased the amounts of flag-TFIIS-bound myc-tagged SPT5, PAF1, and RPBs (Fig. 4E and Supplementary Fig. 5F; lanes 4 and 5); these levels were further enhanced by β-catenin-S33Y (lanes 7 and 8).

### Contribution of NELF to controlling *LGR5* expression cooperatively with stable β-catenin

**Fig. 5:**
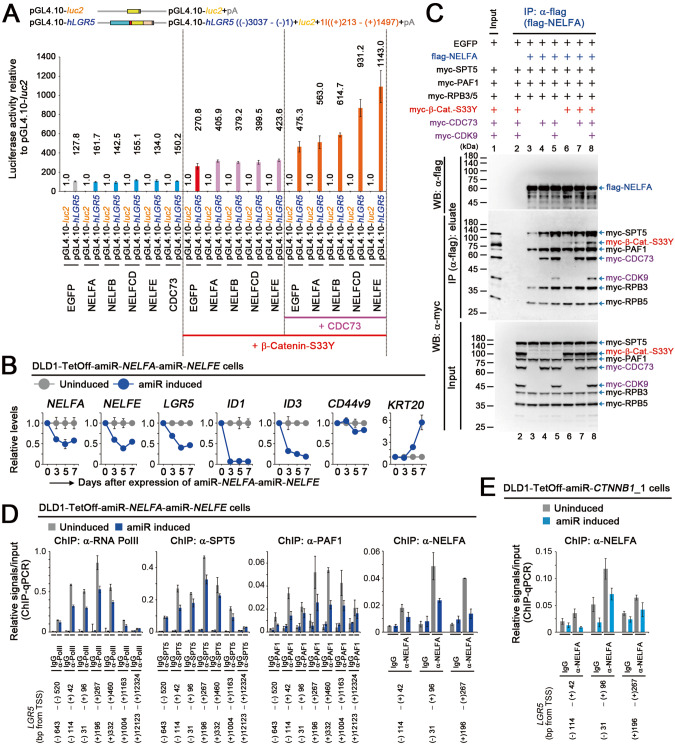
Contribution of NELF to controlling *LGR5* expression cooperatively with stable β-catenin. **A** Bar graph showing the luciferase activity (mean ± SD) of *LGR5 luc* reporter relative to that of pGL4.10-*luc2* control upon expression of the respective NELF molecules with β-catenin-S33Y and CDC73. Red and pink lines at the bottom indicate the additional expression of β-catenin-S33Y and CDC73, respectively. Luciferase activity was determined at 40 h after the *luc* reporter transfection. **B** Line graphs of the qPCR data showing the relative expression levels (mean ± SD) of *NELFA*, *NELFE*, and cancer stemness-related genes upon expression of both amiR-*NELFA* and amiR-*NELFE* (blue) in DLD1-TetOff cells for the indicated days, compared with those in cells not expressing the amiRNAs (gray). **C** Immunoblot images of the IP assays showing the effects of β-catenin-S33Y and CDC73 on the complex formation of flag-NELFA containing SPT5, PAF1, and RPBs. The cells were harvested on day 2 after transfection of the plasmid vectors that expressed the indicated proteins into 293 T cells. After IP of flag-NELFA, the amounts of the co-immunoprecipitad myc-tagged molecules were analyzed via immunoblotting. **D** Bar graphs of the ChIP-qPCR data showing the relative occupancy levels (mean ± SD) of Pol II, SPT5, PAF1, and NELFA on the indicated positions of the *LGR5* gene upon expression of both amiR-*NELFA* and amiR-*NELFE* (blue) in DLD1-TetOff cells for 72 h, compared with those in cells not expressing the amiRNAs (gray). **E** Bar graph of the ChIP-qPCR data showing the relative occupancy level (mean ± SD) of NELFA on the indicated positions of the *LGR5* gene upon expression of amiR-*CTNNB1*_1 (blue) in DLD1-TetOff cells for 24 h, compared with that in cells not expressing the amiRNA (gray).

The NELF complex inhibits transcriptional elongation by Pol II [11, 20], and its interaction with the Pol II complex is mutually exclusive to its interaction with PAF1C, according to in vitro experiments [20]. As our results suggested that NELF contributed to the active Pol II complex formation (Fig. 4), we analyzed the role of NELF in controlling *LGR5* expression. Although all four NELF subunits increased the *LGR5 luc* reporter activity, NELFE induced the highest increase in the *LGR5 luc* reporter activity among the four NELF subunits when co-expressed with β-catenin-S33Y and CDC73 (Fig. 5A). To confirm this, we constructed the DLD1-TetOff cell clones that conditionally expressed an amiRNA against *NELFE*. Inducible knockdown of *NELFE* reduced the expression levels of *LGR5* or *ID1*; however, the reproducibility of these results was not high (data not shown). Hence, we further constructed DLD1-Dual-TetOff cells that inducibly expressed amiRNAs against *NELFA* and *NELFE* (Fig. 5B). Upon expression of the amiRNAs for 5 days, *NELFA* and *NELFE* expression levels decreased to 35–50% of those in uninduced cells (Fig. 5B). Knockdown of both *NELFA* and *NELFE* inhibited the proliferation of the TetOff cells and reduced the expression levels of most cancer stemness-related genes including *LGR5* and *ID1*, whereas *KRT20* and *MUC2* expression levels increased (Fig. 5B and Supplementary Fig. 6A, B), suggesting that both NELF and PAF1C contribute to *LGR5* expression and maintenance of colon cancer stemness. Notably, knockdown of *NELFA* and *NELFE* decreased *ZNFRF3* expression level; however, it did not decrease that of *PHLDA1*, suggesting that the knockdown of *NELFA* and *NELFE* did not completely recapitulate the effect of PAF1 inhibition.

**Fig. 6:**
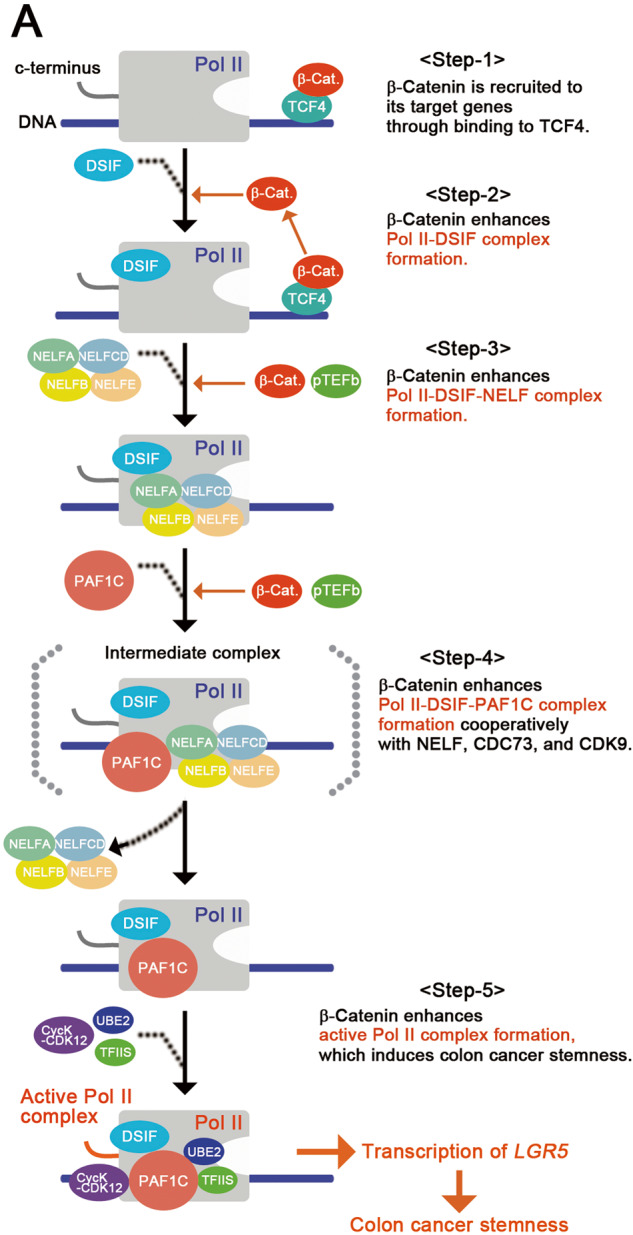
NELF and PAF1 complexes are the core transcriptional machineries that control colon cancer stemness. **A** Illustration showing a hypothetical model of a core transcriptional mechanism that controls colon cancer stemness. Stable β-catenin is recruited to its target genes through TCF4 and stimulates active Pol II complex formation by facilitating the recruitment of NELF to the Pol II complex; it subsequently stimulates the formation of the Pol II–DSIF–PAF1C complex. The NELF complex also contributes to the interaction of PAF1C with Pol II, suggesting that the intermediate complex contains them.

To analyze the role of stable β-catenin in the formation of Pol II–DSIF–NELF complex, we expressed flag-NELFA with myc-tagged SPT5, PAF1, and RPBs. We found that flag-NELFA-bound SPT5 and PAF1 levels increased on expression of NELFE and CDC73 cooperatively with CDK9 (Fig. 5C and Supplementary Fig. 6C; lanes 4 and 5); these levels were further enhanced by β-catenin-S33Y (lanes 7 and 8), suggesting that stable β-catenin enhances the formation of the Pol II–DSIF–NELF complex and the association of NELF with PAF1C cooperatively with NELFE, CDC73, and CDK9. Consistent with this interpretation, we observed that the occupancy levels of Pol II, SPT5, and PAF1 decreased on the *LGR5* gene upon knockdown of *NELFA* and *NELFE* (Fig. 5D). Notably, the occupancy level of NELFA on the *LGR5* gene also decreased upon knockdown of *CTNNB1* (Fig. 5E). Based on these results, we proposed a transcriptional model where stable β-catenin controls colon cancer stemness and *LGR5* expression through the enhancement of active Pol II complex formation by facilitating the formation of the Pol II–DSIF–NELF complex and the subsequent formation of the Pol II–DSIF–PAF1C complex (Fig. 6A).

### Suppression of colon cancer stemness by CDK12 inhibitors

**Fig. 7:**
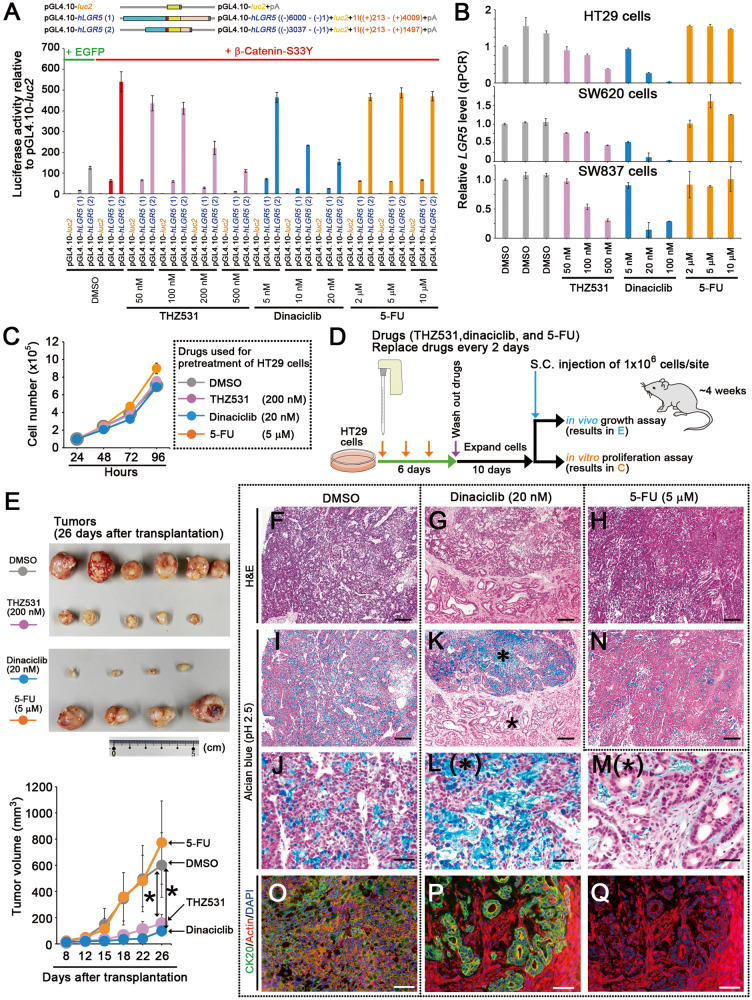
Suppression of colon cancer stemness by CDK12 inhibitors. **A** Bar graph showing the luciferase activity (mean ± SD) of *LGR5 luc* reporter relative to that of pGL4.10-*luc2* control in 293 T cells upon 24-h treatment with THZ531 (pink), dinaciclib (blue), or 5-FU (yellow) at the indicated concentrations. DMSO was used as the control. Green and red lines indicate the expression of EGFP and β-catenin-S33Y, respectively. **B** Bar graphs of the qPCR data showing the relative expression level (mean ± SD) of *LGR5* upon treatment of human colon cancer cells HT29 (top), SW620 (middle), and SW837 (bottom) with THZ531, dinaciclib, or 5-FU at the indicated concentrations for 24 h, compared with that in the DMSO-treated cells (gray). **C** Line graph showing the in vitro proliferation rate of HT29 cells pretreated with the indicated drugs. The In vitro proliferation was analyzed on a culture dish from day 10 after withdrawal of the drugs, compared with that of the DMSO-pretreated cells (gray). **D** Illustration showing the analysis of the effects of THZ531, dinaciclib, and 5-FU on the in vivo tumor growth of HT29 cells. After HT29 cells were treated with the indicated drugs for 6 days, the drugs were washed out. After the CDK-inhibitor-treated cells were cultured without the inhibitors for 10 subsequent days, the in vitro proliferation (**C**) and in vivo growth (**E**) were analyzed on a culture dish and via subcutaneous transplantation into immunodeficient mice, respectively. **E** Dissection micrographs (top) showing the tumors of subcutaneously-transplanted HT29 cells on day 26 following subcutaneous transplantation. A line graph (bottom) showing the tumor volumes (mean ± SD from 4 to 8 tumors). HT29 cells were transplanted into immunodeficient mice on day 10 after withdrawal of the drugs. *P < 0.05, compared with tumors originated from the DMSO-pretreated cells (gray). **F**–**Q** Optical micrographs showing the tumor tissue sections of subcutaneously transplanted HT29 cells pretreated with DMSO (**F**, **I**, **J**, **O**), dinaciclib (**G**, **K**–**M**, **P**, **Q**), or 5-FU (**H**, **N**). H&E (**F**–**H**) and alcian blue stain (pH 2.5; **I**–**N**). Fluorescence micrographs (**O**–**Q**) showing the tumors stained for CK20 (green) and actin (red) with DAPI (blue). **J** is the magnified image of **I**, and **L** and **M** are the magnified images of the areas indicated by asterisks in **K**. Scale bars, 200 μm.

We hypothesized that chemotherapeutic inhibition of the Pol II-related PAF1C function suppressed colon cancer stemness. We tested this hypothesis using THZ531 (a specific inhibitor of CDK12/13 [21]) and with dinaciclib (an inhibitor of multiple transcriptional CDKs, including CDK9 and CDK12/13 [22]). We analyzed the effects of treatment with these two CDK inhibitors and 5-fluorouracil (5-FU; a pyrimidine analog used as an anti-cancer drug for colon cancer) on colon cancer stemness. Both THZ531 and dinaciclib strongly suppressed *LGR5 luc* reporter activity but 5-FU did not (Fig. 7A). Furthermore, colon cancer cells, namely, HT29, SW620, and SW837 when treated with dinaciclib showed decreased the expression levels for *LGR5*, *ID1*, and *CD44v9* (Fig. 7B and Supplementary Fig. 7A). THZ531 treatment reduced the expression level of *LGR5* in HT29 and SW620 cells but did not result in any changes in that of *ID1*. These results are consistent with those observed for *CCNK* knockdown (Fig. 3E).

Then, we analyzed the effects of these CDK inhibitors on in vivo tumor formation. Highly transplantable colon cancer HT29 cells were treated with 200 nM of THZ531, 20 nM of dinaciclib, or 5 μM of 5-FU for 6 days, and a suppression of their proliferation was observed (Supplementary Fig. 7B). However, on day 10 after withdrawal of the drugs, THZ531- and dinaciclib-pretreated cells re-grew in a similar manner to DMSO-pretreated cells (Fig. 7C). Then, they were transplanted subcutaneously into immunodeficient mice (Fig. 7D). The in vivo growth rate of tumors originating from the cells pretreated with dinaciclib (dinaciclib-tumors) or THZ531 (THZ531-tumors) was slower than those of tumors originating from cells pretreated with DMSO or 5-FU (DMSO- and 5-FU-tumors, respectively; Fig. 7E).

Compared with the DMSO-tumors (Fig. 7F and Supplementary Fig. 8A) and 5-FU-tumors (Fig. 7H), the induction of two types of differentiated cells was observed in the THZ531- and dinaciclib-tumors (Fig. 7G and Supplementary Fig. 8B, C). One showed a higher number of cells stained with alcian blue (Fig. 7K, L and Supplementary Fig. 8D; compared with Fig. 7I, J, N), which indicates the induction of goblet cell differentiation, whereas the other showed more branched glandular formation with a polarized characteristic (Fig. 7M, P, Q; compared with Fig. 7O) [23], which is one of characteristics of well-differentiated colon cancer cells. Notably, the expression level of *LGR5* in THZ531- and dinaciclib-tumors was lower than that in DMSO-tumors (Supplementary Fig. 8E). In consistent with this result, TOPflash reporter activity in the cultured HT29 cells derived from THZ531- and dinaciclib-tumors was lower than that in the cells derived from DMSO-tumors (Supplementary Fig. 8F). This finding suggests that these CDK inhibitors suppress cancer stemness and induce the differentiation of colon cancer cells; moreover, these effects are retained after the withdrawal of the CDK inhibitors.

## Discussion

We showed that the expression of PAF1C and NELF components increased the *LGR5 luc* reporter activity upon simultaneous expression of stable β-catenin (Figs. 2–5). Additionally, stable β-catenin promoted the formation of the Pol II–DSIF–NELF and Pol II–DSIF–PAF1–TFIIS complexes (Figs. 4 and 5). Crucially, the suppression of PAF1C components or NELF reduced the expression levels of most β-catenin-targeted genes that are required for the maintenance of colon cancer stemness (Figs. 2, 3 and 5); this also suppressed the in vivo tumor growth of colon cancer cells (Fig. 2). These results collectively suggest that NELF and PAF1 complexes are the transcriptional machineries that control colon cancer stemness by integrating the input from stable β-catenin to control gene expression (Fig. 6A). Notably, PAF1 contributes to the maintenance of pancreatic cancer stemness via its interaction with PHF5A and DDX3 [24], but not through the typical PAF1 complex, Pol II pause release, or stable β-catenin. These findings suggest that PAF1 regulates the stemness of several types of cancer cells through different mechanisms.

NELF stabilizes the Pol II–DSIF complex and competes with PAF1C for binding to Pol II, whereas CDK9-mediated phosphorylation of NELF promotes the subsequent binding of PAF1C to the Pol II−DSIF complex [20, 25]. Our results showed that NELF contributed to the active Pol II complex formation and *LGR5* expression, which was enhanced by stable β-catenin cooperatively with CDK9 and CDC73 (Figs. 4 and 5). Thus, the Pol II–DSIF–NELF complex induces Pol II pausing and can act as a prerequisite for the formation of the Pol II–DSIF–PAF1C complex. Therefore, NELF facilitates the formation of Pol II–DSIF–PAF1C complex upon receiving the inputs of CDK9 and stable β-catenin (Fig. 6A).

Cancer stemness was suppressed by dinaciclib or THZ531 treatment (Fig. 7), or knockdown of *CCNK* (Fig. 3); hence, CDK12 inhibitors are potential chemotherapeutic agents against colon cancer. Dinaciclib decreased *LGR5* and *ID1* expression levels and exhibited higher tumor growth suppressing effects than that of THZ531 (Fig. 7). These results suggest that the combinational inhibition of some transcriptional CDKs, including that of CDK9 and CDK12, is a potentially effective strategy to treat colon cancer.

## Materials and methods

### Cells and TetOff clones

Cells were cultured in Dulbecco’s Modified Eagle’s Medium supplemented with 5% fetal bovine serum. To generate TetOff cell clones, plasmids were transfected using Lipofectamine LTX^TM^ reagent with Plus^TM^ reagent. TetOff cell clones of the representative human colon cancer cells DLD1 (CCL-221; ATCC) and LS174T (CL188; ATCC: RRID:CVCL_1384) were generated using pTetOff (631017; TAKARA) and pTRE-Tight (631059; TAKARA)-based plasmid vectors as previously described [26]. Doxycycline (24390-14-5; TGI), a tetracycline analog, was added to culture media at a final concentration of 0.5 µg/mL.

### Supplementary information

Supplemental information

## Data Availability

All relevant data are available from the authors upon request.
