# Supplementary material for: NELF and PAF1C complexes are core transcriptional machineries controlling colon cancer stemness
Source: Oncogene. 2024 Jan 5;43(8):566–77. doi: 10.1038/s41388-023-02930-0 (PMC10873196; doi:10.1038/s41388-023-02930-0)
Supplement: Supplementary file 1 — Supplemental information [file 41388_2023_2930_MOESM1_ESM.docx]

Supplementary Information for:

NELF and PAF1C complexes are core transcriptional machineries controlling colon cancer stemness

Koji Aoki^1^, Akari Nitta^1^, and Ayumi Igarashi^1^

^1^ Department of Pharmacology, Faculty of Medicine, University of Fukui, Fukui, Japan.

Supplementary Materials and Methods

Supplementary References

and Supplementary Figures 1-8 and Legends.

**Supplementary Materials and Methods**

**Reagents**

Reagents used in this study are listed below.

| **Reagents used in this study** | | | |
| --- | --- | --- | --- |
| **Reagents** | **Cat. No.** | **Suppliers** | **Applications** |
| Doxycycline | 24390-14-5 | TGI | Cell culture |
| Lipofectamine LTXTM reagent with PlusTM reagent | 15338100 | ThermoFisher Scientific | Generation of TetOff cells |
| Polyethylenimine MAXTM | 24765-100 | Polysciences | Generation of TetOff cells |
| pTetOff | 631017 | TAKARA (Clontech) | Generation of TetOff cells |
| pTRE-Tight | 631059 | TAKARA (Clontech) | Generation of TetOff cells |
| Hygromycin-resistance gene | 631625 | TAKARA (Clontech) | Cell culture |
| Puromycin-resistance gene | 631626 | TAKARA (Clontech) | Cell culture |
| pBlueScript II phagemid vector |  | Agilent | Plasmid construction |
| pcDNA6 | Invitrogen V22020 | Fisher Scientific | Plasmid construction |
| pGL4.10 [luc2] vector | E6651 | Promega | Luciferase reporter assay |
| pGL4.75 [hRuc/CMV] vector | E6931 | Promega | Luciferase reporter assay |
| NucleoBond® Xtra Midi Plus | 740412.50 | MNA | Plasmid purification |
| Hygromycin B gold | ant-hg | InvivoGen | Cell culture |
| Puromycin | ant-pr | InvivoGen | Cell culture |
| G418 | 09380-44 | nacalai tesque | Cell culture |
| PrimeSTAR^®^ GXL DNA polymerase | R050 | TAKARA | Plasmid construction |
| KOD-Plus-Neo | KOD401 | TOYOBO | Plasmid construction |
| TRI Reagent^®^ | T9424 | Sigma-Aldrich | RNA extraction |
| RNeasy Midi kit^®^ | 75144 | QUIAGEN | RNA purification |
| FastGene™ RNA Basic Kit | FG-80250 | Nippon Genetics | RNA purification |
| DNase I | 314-08071 | NipponGene | RNA purification |
| ReverTra Ace | TRT-101 | TOYOBO | ssDNA synthesis |
| AMPure XP | A63881 | Beckman coulter | Purification of ssDNA |
| RNAClean XP | A63987 | Beckman coulter | Purification of ssDNA |
| QuantiFluor^®^ ssDNA system | E3190 | Promega | Determination of ssDNA concentration |
| KOD SYBER^®^ qPCR mix | QKD-201 | TOYOBO | qPCR assay |
| Formaldehyde | 064-03843 | FUJIFILM | ChIP and tissue fixation |
| Micrococcal nuclease | NFCP | Worthington Bio. Corp. | ChIP assay |
| RNase A | RPDF | Worthington Bio. Corp. | ChIP assay |
| 3xFLAG peptide | F4799 | Sigma-Aldrich | ChIP and IP assays |
| 3xFLAG peptide | GEN-3-FLAG | Protein Ark | ChIP and IP assays |
| Protein G sepharose 4 Fast Flow | 17-0618-01 | GE Healthcare | ChIP assay |
| Protein A/G PLUS-Agarose: | sc-2003 | Santa Cruze | ChIP assay |
| Proteinase K | 4548995075352 | FUJIFILM | ChIP assay |
| Phenol | 4987481307285 | FUJIFILM | ChIP assay |
| Chloroform | 038-02606 | FUJIFILM | ChIP assay |
| Dual-Luciferase Reporter Assay System | E1910 | Promega | Luciferase reporter assay |
| THZ531 | S6595 | Selleck | Treatment of cancer cells |
| Dinaciclib | S2768 | Selleck | Treatment of cancer cells |
| 5-Fluorouracil | 064-01403 | FUJIFILM | Treatment of cancer cells |
| Dimethyl Sulfoxide | 064-01403 | FUJIFILM | Drug solvent |
| BD Matrigel Matrix | 354234 | ThermoFisher Scientific | Organoid culture and Transplantation exp. |
| Sucrose | 4987481323742 | FUJIFILM | Transplantation exp. |
| GM Hematoxylin stain solution for HE staining | 30082 | Muto pure chemicals | Pathological analysis |
| Pure Eosin solution | 32043 | Muto pure chemicals | Pathological analysis |

**Antibodies**

Antibodies used in this study are listed below.

| **Antibodies used in this study** | | | | |
| --- | --- | --- | --- | --- |
| **Antibodies** | **Clones** | **Cat. No.** | **Suppliers** | **Applications** |
| Anti DYKDDDDK tag Antibody Beads |  | 018-22783 | FUJIFILM | ChIP, IP |
| Rabbit IgG−Agarose saline suspension |  | A2909 | Sigma-Aldrich | ChIP |
| Anti-beta-catenin antibody |  | 610154 (RRID:AB_397555) | BD Transduction | ChIP, IHC |
| ANTI-FLAG® M2 Agarose Affinity Gel |  | A2220RRID (RRID: AB_10063035) | Sigma-Aldrich | ChIP, IP |
| Goat Anti-Rabbit IgG(H+L)-HRP Conjugate |  | 1706515 (RRID:AB_11125142) | Bio-Rad | ChIP |
| Anti-Mouse IgG (whole molecule) −Agarose antibody produced in goat |  | A6531 (RRID:AB_258295) | Sigma-Aldrich | ChIP, IP |
| Anti β-Actin, Monoclonal Antibody |  | 013-24553 | FUJIFILM | WB, IHC |
| Cy5 AffiniPure Goat Anti-Rabbit IgG (H+L) (min X Hu,Ms,Rat Sr Prot) |  | 111-175-144 (RRID:AB_2338013) | Jackson Immuno Research Laboratories, Inc. | IHC |
| Anti-RNA polymerase II antibody | CTD4H8 | 05-623 (RRID:AB_309852) | Merck | ChIP |
| Normal mouse IgM |  | sc-3881 (RRID:AB_737292) | SANTA CRUZ | ChIP |
| Anti-Myc-tag mAb |  | M192−3 (RRID:AB_11160947) | MBL | WB |
| Anti-Pol II antibody | F-12 | sc-55492 (RRID:AB_10708864) | SantaCruze Biotechnology | ChIP |
| Anti-RNA polymerase II subunit B1 Antibody | 4F8 | ABE30 (RRID:AB_10846922) | Merck | ChIP |
| Normal Rat IgG Whole Molecule, Purified |  | 147-09521 | FUJIFILM | ChIP |
| Anti-SPT5 antibody | D-3 | sc-133217 (RRID:AB_2196394) | SantaCruze Biotechnology | ChIP |
| Anti-NELF-A antibody | G-11 | sc-365004 (RRID:AB_10708864) | SantaCruze Biotechnology | ChIP |
| Ubiquityl-Histone H2B(Lys120)(D11)XP Rabbit mAb |  | 5546S (RRID:AB_10693452) | Cell Signaling | WB |
| Goat Anti-Mouse IgG (H+L) HRP Conjugate |  | 1706516 (RRID:AB_11125547) | Bio-Rad | WB |
| Anti-PAF1/PD2 antibody - ChIP Grade |  | ab20662 (RRID:AB_2159769) | Abcam | ChIP |
| Anti-Cytokeratin 20 antibody |  | ab76126 (RRID:AB_1310117) | Abcam | Pathological analysis |

**TetOff cell clones**

Cells were cultured in Dulbecco's Modified Eagle's Medium supplemented with 5% fetal bovine serum. To generate TetOff cell clones, plasmids were transfected using Lipofectamine LTX^TM^ reagent with Plus^TM^ reagent. TetOff cell clones of the representative human colon cancer cells DLD1 (CCL-221; ATCC) and LS174T (CL188; ATCC) were generated using pTetOff (631017; TAKARA) and pTRE-Tight (631059; TAKARA)-based plasmid vectors as previously described [1]. Doxycycline (24390-14-5; TGI), a tetracycline analog, was added to culture media at a final concentration of 0.5 µg/mL. The DLD1-TetOff or LS174T-TetOff cells were transfected with pTRE-Tight-based plasmid vectors inducibly expressing UBE2A mutants, and artificially-designed microRNAs (amiRNA) against *PAF1*, *CTNNB1*, *CDC73*, *CTR9*, and *CCNK*. The transfected TetOff cells were cultured in the presence of 200 µg/mL hygromycin B gold (ant-hg; InvivoGen) for more than 2 weeks. Single-cell clones were then expanded. To generate DLD1-Dual-TetOff cells inducibly expressing amiRNAs against *NELFA* and *NELFE*, we first generated the Single-TetOff cells that expressed an amiRNA against *NELFE*, using a hygromycin-resistance gene (631625; TAKARA) and then introduced into the TetOff-amiR-*NELFE* cell clone a plasmid containing an amiRNA against *NELFA*, using a puromycin-resistance gene (631625; TAKARA). We established stable TetOff cell clones by culturing the cells in the presence of 400 µg/mL G418 (09380-44; nacalai tesque), 200 µg/mL hygromycin B gold, or 1 µg/mL puromycin (ant-pr; InvivoGen). After the several cell clones were expanded, the expression levels of *NELFA* and *NELFE* were confirmed using the qPCR analysis.

**Chromatin immunoprecipitation analysis**

For the preparation of cells used for the ChIP analysis, the expression of amiRNAs was induced by withdrawing DOX from the culture media at the same time as they were spread onto a 150-mm dish (5–7.5 × 10^6^ cells per dish). At the indicated hours after induction of amiRNAs expression in DLD1-TetOff cells, the cells were fixed in 15 mL of 1% formaldehyde in PBS for 10 min at 25 °C with gentle rotation using a temperature-controlled shaker. After 1.66 mL of 1.25 M glycine was added to the fixation solution, the cells were incubated for 5 min at 25 °C with gentle rotation in the shaker. After washing twice with 15 mL of ice-cold PBS, the cells were scraped with 1 mL of ice-cold PBS using a cell scraper and transferred into a 1.5-mL tube. After centrifugation at 8,000 rpm (5,800 g; MX-307, TOMY) for 1 min, the supernatants were discarded, and cells were resuspended in 950 µL of the lysis buffer (150 mM NaCl, 50 mM Tris-HCl, 10 mM EDTA, and proteinase inhibitors) and incubated at 10 °C to prevent precipitation of SDS. After pipetting the samples five times, 105 µL of 1% SDS was added to the lysate and mixed by pipetting carefully. After incubating the lysates at 10 °C for 10 min, they were sonicated using a Covaris Focused-ultrasonicator M220 (Covaris) to shear DNA to fragment sizes of 200–1000 base pairs (setting parameters; minimum, 5.0 °C, set point, 7.0 °C, maximum, 9.0 °C; peak power, 75; duty factor, 10; cycle/burst, 200; duration, 1200 s). The lysates were then centrifuged at 15,000 rpm (20,400 g; MX-307, TOMY) for 15 min at 8 °C, and the supernatants were transferred into a new tube. Protein concentrations of the lysates were determined by the Bradford protein assay. After the protein concentrations of the lysates were equalized, the lysates were diluted five times in the ChIP dilution buffer (150 mM NaCl, 20 mM Tris-HCl, 1% Triton X-100, 2 mM EDTA). Equal amounts of lysates were then used for immunoprecipitation assays. Aliquots of lysates were also used as input DNA.

After adding primary antibodies to the diluted lysates, they were incubated overnight at 4 °C with rotation. The blocked protein A/G beads were added to the samples and incubated for an additional 3 h at 4 °C with rotation. After washing the beads five times with dilution buffer and once with TE buffer, the elution buffer (0.5% SDS, 50 mM Tris-HCl, and 5 mM EDTA) containing an RNase was added to the beads. The RNase was also added to the input DNA. The samples were reverse-crosslinked at 65 °C overnight and treated with a proteinase K. After centrifugation, the supernatant was transferred to a new tube. ChIP-DNA was purified by the standard phenol-chloroform extraction method and analyzed via qPCR. The qPCR signals obtained from the ChIP-DNA were normalized to those from the input DNA. Experiments were repeated at least twice. The oligonucleotide primer sequences used for the ChIP-qPCR analysis are listed above.

**Gene cloning**

The open reading frames of human *CTNNB1*, *PAF1*, *CTR9*, *LEO1*, *CDC73*, *RTF1*, *RNF20*, *RNF40*, *WDR61*, *UBE2A*, *TCEA2*, *CCNK*, *CDK12*, *CDK9*, *NELFA*, *NELFB*, *NELFCD*, *NELFE*, *SPT5*, *RPB2*, *RPB3*, *RPB5,* and *RPB8* genes were amplified via PCR and cloned into the pBlueScript II phagemid vector (212207; Agilent). PCR was carried out using PrimeSTAR^®^ GXL DNA polymerase (R050; TAKARA) or KOD-Plus-Neo DNA polymerase (KOD401; TOYOBO) using complementary DNA (cDNA) synthesized from RNA isolated from 293T cells (CRL-3216; ATCC) or colon cancer cell lines (DLD1 or LS174T) as a template. The cloned cDNA was fused with 3 x flag or 3 x myc tags at the N-terminus using a pcDNA6/V5-His A (V220-01; Invitrogen) -modified plasmid expression vector. The oligonucleotide primer sequences used for the plasmid construction are listed below. The lowercase letters in the oligonucleotides indicate complementary sequences, whereas the uppercases indicate non-complementary sequences required for plasmid construction.

| **Oligonucleotide primers used for cloning genes in this study** | | | |
| --- | --- | --- | --- |
| **Genes** | **Primer names** | **Sequences** | **Application** |
| *RNF20* | hRNF20-F | ATGCATGCGGATCCatgtcaggaattggaaataaaagagc | Gene cloning |
|  | hRNF20-R | GCATGCATCTCGAGCTTAAGtcaaccaatgtagatgcgatg | Gene cloning |
| *RNF40* | hRNF40-F | ATGCATGCGGATCCatgtctgggccaggcaacaaacgcg | Gene cloning |
|  | hRNF40-R | GCATGCATGAATTCtcagctgatgtagatacgatggaag | Gene cloning |
| *UBE2A* | hUSB2A-F | ATGCATGCAAGCTTACCGGTatgtccaccccggctcggcggcg | Gene cloning |
|  | hUSB2A-R | GCATGCATCTCGAGCTTAAGtcaacaatcacgccagctttgttc | Gene cloning |
| *RPB2* | hRPB2-F | ATGCATGCGGTACCatgtacgacgcggatgaggatatgc | Gene cloning |
|  | hRPB2-R | GCATGCATCTCGAGctaaacactcatcattcgcggtgc | Gene cloning |
| *RPB8* | hPOLR2H-F | ATGCATGCGGATCCatggcgggcatcctgtttgagg | Gene cloning |
|  | hPOLR2H-R | GCATGCATGAATTCtcaggcgaggttcagaaggc | Gene cloning |
| *RPB5* | hPOLR2E-F | ATGCATGCGAATTCatggacgacgaggaggagacgtacc | Gene cloning |
|  | hPOLR2E-R | GCATGCATCTCGAGctactgcaccagccggtagg | Gene cloning |
| *RPB3* | hPOLR2H-F | ATGCATGCGGATCCatgccgtacgccaaccagcctaccg | Gene cloning |
|  | hPOLR2H-R | GCATGCATAAGCTTttaatttatggttagcacatcactctgg | Gene cloning |
| *PAF1* | hPAF1-F | ATGCATGCGGATCCatggcgcccaccatccagacccagg | Gene cloning |
|  | hPAF1-R | GCATGCATGAATTCtcagtcactgtcactatcagcttcac | Gene cloning |
| *CTR9* | hCtr9-F | ATGCATGCACCGGTatgtcgcggggctccatcgagattcccctccggg | Gene cloning |
|  | hCtr9-R | GCATGCATGTCGACCTTAAGctagtcactatcatctgacccatgttctg | Gene cloning |
| *LEO1* | hLeo1-F | ATGCATGCACCGGTGGATCCatggcggatatggaggatctcttcgggag | Gene cloning |
|  | hLeo1-R | GCATGCATCTCGAGCTTAAGtcaatcatcatcttcttcctcttcatcgc | Gene cloning |
| *RTF1* | hRTF1-F2 | ATGCATGCACCGGTatgcgcggtcgcctttgtgtgggtcgagc | Gene cloning |
|  | hRTF1-R2 | GCATGCATCTCGAGCTTAAGtcaaataagccctcgtcgttttttgtagtc | Gene cloning |
| *CTNNB1* | hCTNNB1-F | ATGCGGATCCATGGCTACTCAAGCTGATTTGA | Gene cloning |
|  | hCTNNB1-R | GCATATCGATTTACAGGTCAGTATCAAACCA | Gene cloning |
| *CDC73* | hCDC73-F | ATGCATGCGAATTCatggcggacgtgcttagcgtcctgcg | Gene cloning |
|  | hCDC73-R | GCATGCATCTCGAGTCAtcagaatctcaagtgcgatttatgc | Gene cloning |
| *CDK12* | hCDK12-F | ATGCATGCACCGGTGGATCCGCCGCCACCatgcccaattcagagagacatggg | Gene cloning |
|  | hCDK12-R | GCATGCATGTCGACCTTAAGttagtaaggaactcctctccctcttc | Gene cloning |
| *NELFE* | hNELFE-F | ATGCATGCGGATCCatgttggtgataccccccggactgag | Gene cloning |
|  | hNELFE-R | GCATGCATGAATTCctagaagccatccacaaggttttc | Gene cloning |
| *CTNNB1* | hCTNNB1-F | ATGCGGATCCATGGCTACTCAAGCTGATTTGA | Gene cloning |
|  | hCTNNB1-R | GCATATCGATTTACAGGTCAGTATCAAACCA | Gene cloning |
| *NELFB* | hNELFB-F | ATGCATGCGGATCCatgctggccgagctggagggcgccggg | Gene cloning |
|  | hNELFB-R | GCATGCATGAATTCtcagagcggggcaggggcgggcac | Gene cloning |
| *SPT5* | hSPT5-F | ATGCATGCGAATTCatgtcggacagcgaggacagc | Gene cloning |
|  | hSPT5-R | GCATGCATCTCGAGtcaggcttccaggagcttccc | Gene cloning |
| *NELFA* | hNELFA-F | ATGCGGATCCatgccggggcagcggcgcgcgc | Gene cloning |
|  | hNELFA-R | GCATGAATTCctaggacacattggtcatgggcttg | Gene cloning |
| *NELFCD* | hNELFCD-F | ATGCGGATCCatgcgccgcgctcgctcgcgggagg | Gene cloning |
|  | hNELFCD-R | GCATGAATTCttagttcaccatgatgaagttagatttgc | Gene cloning |
| *TECA2* | mTECA2-F | ATGCGGATCCatgggcaaggaggaagagattgctcg | Gene cloning |
|  | mTECA2-R | GCATGAATTCTCAggagctacgggtctgcacctg | Gene cloning |

**Mutagenesis of UBE2A**

The mutants of UBE2A were constructed via PCR of the wild-type UBE2A as a template with the oligonucleotide primers listed below.

| **Oligonucleotide primers used for construction of UBE2A mutants** | | | |
| --- | --- | --- | --- |
| **Mutants** | **Primer names** | **Sequences** | **Application** |
| UBE2A-C88S | hUBE2A-C88S-F | AGTATAtctCTGGACATACTTCAGAACCGT | Mutagenesis |
|  | hUBE2A-C88S-R | GTCCAGAGATATACTACCATCTGCATAGAC | Mutagenesis |
| UBE2A-C88V | hUBE2A-C88V-F | AGTATAGTTCTGGACATACTTCAGAACCGT | Mutagenesis |
|  | hUBE2A-C88V-R | GTCCAGAACTATACTACCATCTGCATAGAC | Mutagenesis |

**Artificial microRNA construction**

The target sequences of artificial microRNAs (amiRNAs) were selected using the i-Score Designer (https://www.med.nagoya-u.ac.jp/neurogenetics/i_Score/i_score.html). The sequences of complementary strands for the amiRNAs and backbone were designed according to the manufacturer’s instruction of the BLOCK-iT™ Pol II miR RNAi Expression Vector Kit (K493500; ThermoFisher Scientific) and an earlier report [2]. EGFP and amiRNAs were inserted into a multi-cloning site in the order of P_Tight_ and bpA, respectively, in the pTRE-Tight plasmid vector (631059; Clontech). Subsequently, amiRNAs were inducibly expressed under the DOX-regulated TetOff system. The oligonucleotide primer sequences used for the amiRNA construction are listed below. Uppercase letters in the oligonucleotides show complementary sequences for the target genes, whereas lowercase letters indicate backbone sequences.

| **List of amiR sequences used in this assay** | | | |
| --- | --- | --- | --- |
| **amiRs** | **Target sequence positions** | **Sequences** | **Application** |
| h*CDC73*-amiR | 98-118 | ttgctgaaggctgtatgctgAGTTGGTCTTCACATTCTTGGgttttggccactgactgacCCAAGAATGAAGACCAACTcaggacacaaggcctgtta | amiRNA  construction |
| h*CTNNB1*-amiR_1 | 652-672 | ttgctgaaggctgtatgctgATGATGGGAAAGGTTATGCAAgttttggccactgactgacTTGCATAATTTCCCATCATcaggacacaaggcctgtta | amiRNA  construction |
| h*CTNNB1*-amiR_2 | 984-1004 | ttgctgaaggctgtatgctgTTTTCGTAAGTATAGGTCCTCgttttggccactgactgacGAGGACCTACTTACGAAAAcaggacacaaggcctgtta | amiRNA  construction |
| h*CTNNB1*-amiR_3 | 402-422 | ttgctgaaggctgtatgctgTTAATCAAGTTTACAACTGCAgttttggccactgactgacTGCAGTTGAACTTGATTAAcaggacacaaggcctgtta | amiRNA  construction |
| h*PAF1*-amiR_3 | 492-513 | ttgctgaaggctgtatgctgTCTTTGTATATTTCTTCCTCGgttttggccactgactgacCGAGGAAGATATACAAAGAcaggacacaaggcctgtta | amiRNA  construction |
| h*PAF1*-amiR_5 | 853-864 | ttgctgaaggctgtatgctgAATTTTGTAGTCATACACATCgttttggccactgactgacGATGTGTAACTACAAAATTcaggacacaaggcctgtta | amiRNA  construction |
| h*NELFA*-amiR | 453-474 | ttgctgaaggctgtatgctgTTTTTGTTCAAGTACTGGCACgttttggccactgactgacGTGCCAGTTTGAACAAAAAcaggacacaaggcctgtta | amiRNA  construction |
| h*CCNK*-amiR | 421-442 | ttgctgaaggctgtatgctgTAAGATTCTCTCCAGAACCATgttttggccactgactgacATGGTTCTAGAGAATCTTAcaggacacaaggcctgtta | amiRNA  construction |
| h*NELFE*-amiR | 624-645 | ttgctgaaggctgtatgctgTCTCTGTCTCTGTCTCGATCCgttttggccactgactgacGGATCGAGAGAGACAGAGAcaggacacaaggcctgtta | amiRNA  construction |
| h*CTR9*-amiR | 3133-3153 | ttgctgaaggctgtatgctgTTTCTTTCGTTGTTCGTCCTCgttttggccactgactgacGAGGACGAAACGAAAGAAAcaggacacaaggcctgtta | amiRNA  construction |

**RT-qPCR analysis**

For the RT-qPCR analysis, RNA was isolated from the cells using TRI Reagent^®^ . After DNase treatment, RNA was purified using FastGene™ RNA Basic Kit. The cDNA was synthesized using a ReverTra Ace and purified using AMPure XP or RNAClean XP. The concentrations of the purified cDNAs were determined using the QuantiFluor^®^ ssDNA system and the GloMax^®^-Multi+ detection system. Equal amounts of cDNAs were used for the qPCR assays using StepOnePlus^TM^, QuantStuido^®^ 3 or 5 (ThermoFisher Scientific) provided by University of FUKUI, Life Science Research Center. qPCR was carried out with a KOD SYBER^®^ qPCR mix (QKD-201; TOYOBO) using the following oligonucleotide primers. Experiments were repeated at least twice.

To analyze the effects of inhibiting CDK12 and CDK13 on the *LGR5* expression, colon cancer cells HT29 (HTB-38; ATCC), SW837 (CCL-235; ATCC), and SW620 (CCL-227; ATCC) were treated with THZ531, dinaciclib, or 5-FU for 24 h at the indicated concentrations (Fig. 7). Then, RNA was purified and *LGR5* expression level was analyzed by qPCR as described above.

To analyze *LGR5* expression in the *in vivo* tumors, RNA was extracted from the tumors transplanted in immunodeficient mice using TRI Reagent^®^. Then, RNA was purified for the cDNA synthesis as described above. To determine the expression level of *LGR5*, that of *β-ACTIN (ACTB)* was used as an internal control to eliminate the contamination of stromal cells derived from the immunodeficient mice. The expression level of *ACTB* was determined using oligonucleotide primers specific to the human *ACTB*.

| **Oligonucleotide primers used for qPCR analysis** | | | | |
| --- | --- | --- | --- | --- |
| **Target genes** | **Oligonucleotide primer names** | **sequences** | **Application** | **References** |
| *CD44* | hCD44pv9F | CAGAGCTTCTCTACATCACA | qPCR | Ref. 4 |
|  | hCD44hs3R | TTTGCTCCACCTTCTTGACTCC | qPCR |  |
| *ID3* | ID3-46F | TGCCTGTCGGAACGCAGTCT | qPCR | This study |
|  | ID3-311R | AGCTCGGCTGTCTGGATGGGAA | qPCR | This study |
| *LGR5* | LGR5-1788F | TGGGGTCATCGCAGCAGTGA | qPCR | This study |
|  | LGR5-2167R | TGGTGCTGGGCTCCCCAAAA | qPCR | This study |
| *SOX9* | SOX9-316F | AAGCGGCCCATGAACGCCTT | qPCR | This study |
|  | SOX9-623R | TGCAGCGCCTTGAAGATGGC | qPCR | This study |
| *ASCL2* | ASCL2-180F | CAACCGCGTGAAGCTGGTGA | qPCR | This study |
|  | ASCL2-296R | ATGTACTCCACGGCTGAGCGCA | qPCR | This study |
| *ZNFRF3* | ZNFRF3-2345F | ATGAAGAGAAGCAGGTGGCC | qPCR | This study |
|  | ZNFRF3-2747R | GTGCTGGACTCCTGAGTGTC | qPCR | This study |
| *RNF43* | RNF43-1929F | AAAATCCAGCCTCTCTGCCC | qPCR | This study |
|  | RNF43-2108R | GGTCCACAGATCAAGGGGTG | qPCR | This study |
| *MYC* | Myc-468F | CAAAGACAGCGGCAGCCCGA | qPCR | This study |
|  | Myc-633R | TTGCGAGGCGCAGGACTTGG | qPCR | This study |
| *EPHB2* | EPHB2-2237F | ACCTGGCTGCCCGCAACATC | qPCR | This study |
|  | EPHB2-2487R | CCAGTAGGGCCGCTCCCCAT | qPCR | This study |
| *ALDH1B* | ALDH1B-673F | ATCAAGGAGGCAGGCTTTCC | qPCR | This study |
|  | ALDH1B-1098R | CTGCTCCTTGTCCACCTGAG | qPCR | This study |
| *BMI1* | hBMI1-37F | AATCCCCACCTGATGTGTGT | qPCR | This study |
|  | hBMI1-435R | CCGATCCAATCTGTTCTGGT | qPCR | This study |
| *KRT20* | KRT20-693F | AGGCCTGAACCTTGGCGTCAT | qPCR | This study |
|  | KRT20-971R | TGGCTGCTGTAACGGGCCTT | qPCR | This study |
| *PROM1* | PROM1-340F | GTCCTGGGGCTGCTGTTTAT | qPCR | This study |
|  | PROM1-472R | AGCATTTCCTCAGGAAGGGC | qPCR | This study |
| *PHLDA1* | PHLDA1-771F | AGAGGGCAAGGAGATCGACT | qPCR | This study |
|  | PHLDA1-1096R | GGTGCGAGTGAGGATGAGAG | qPCR | This study |
| *ID1* | ID1-189F | CGTGCTGCTCTACGACATGA | qPCR | This study |
|  | ID1-339R | GGATTCCGAGTTCAGCTCCA | qPCR | This study |
| *MUC1* | hMUC1-465F | AGACGTCAGCGTGAGTGATG | qPCR | This study |
|  | hMUC1-636R | CAGCTGCCCGTAGTTCTTTC | qPCR | This study |
| *MUC2* | MUC2-12884-12901F | AACCCACACCGCCCCTGC | qPCR | Ref. 5 |
|  | MUC2-13256‑13237R | ACGGCCCCGTTAAGCACAGC | qPCR |  |
| *MUC3A* | hMUC3A-8794F | ACCCTTACATCACGCAGGAC | qPCR | This study |
|  | hMUC3A-8957R | CATCTGGTCTGGAGCTGACA | qPCR | This study |
| *MUC4* | hMUC4-2740F | AGAACCAGCAGAGGGTCTGA | qPCR | This study |
|  | hMUC4-2869R | GAGTGTGGGTCTcGGTTTGT | qPCR | This study |
| *MUC5* | hMUC5B-15200F | TCCACTATGAGTGCGAGTGC | qPCR | This study |
|  | hMUC5B-15326R | AAGCGTGCATGGATCTCTCT | qPCR | This study |
| *MUC6* | hMUC6-1864F | GCCTGCAACTACGAGGAGAC | qPCR | This study |
|  | hMUC6-1986R | GATGGTGCAGTTGTCCACAC | qPCR | This study |
| *MUC13* | hMUC13-44F | TAAACACAGCCACCAACCAA | qPCR | This study |
|  | hMUC13-213R | GGGAGCAGGTGAAGTAGCTG | qPCR | This study |
| *MUC17* | hMUC17-12986F | TAGTGGAGTACCGGGACCAG | qPCR | This study |
|  | hMUC17-13268R | TGCCGTTTCACCTCTCTCTT | qPCR | This study |
| *SPDEF* | hSPDEF-460F | AATGTGCAGAAGTGGCTCCT | qPCR | This study |
|  | hSPDEF-644R | CAGGTGAAGTCCGCTCTTTC | qPCR | This study |
| *SPT4* | hSPT4-216F | CAAGTGGCAGCGAGTCAGTA | qPCR | This study |
|  | hSPT4-320R | GCCACTCCTCGACTTTTCAG | qPCR | This study |
| *SPT6* | hSPT6-3283F | AACCCAGAGCGACTGAAAGA | qPCR | This study |
|  | hSPT6-3444R | CTCCTCTGTGTTGGGAGAGC | qPCR | This study |
| *CTNNB1* | hCTNNB1-2013F | gaaaCGGCTTTCAGTTGAGC | qPCR | This study |
|  | hCTNNB1-2178R | CTGGCCATATCCACCAGAGT | qPCR | This study |
| *CTR9* | hCTR9-2842F | GAAGATGAGGAGGGTGGTGA | qPCR | This study |
|  | hCTR9-2965R | CTTTTGGTGGACGTCGTTTT | qPCR | This study |
| *CDC73* | CDC73-297F | TGGTGAAGCGTCAACATCGGCAAGT | qPCR | This study |
|  | CDC73-475R | CCTCCAAACGGGCAGCCAATCTCTC | qPCR | This study |
| *NELFA* | NELFA-416F | TGGGTGAGTGTGAAGCGTCTGCCAT | qPCR | This study |
|  | NELFA-683R | ATGCCTTTGAGTGGGGTGGTGGTGT | qPCR | This study |
| *NELFE* | NELFE-176F | CCACAGCAACAGAGCAGGCAAAGCA | qPCR | This study |
|  | NELFE-334R | TCTGGAACGGCTGGAAAGTGGGGA | qPCR | This study |
| *PAF1* | PAF1-940F | ATCTTCCGAGAGGGTGACGGGGTTT | qPCR | This study |
|  | PAF1-1117R | TTTCTAGCTGGGCCTTCCGTGCCT | qPCR | This study |
| *CTNNB1* | CTNNB1-1414F | ACCAGCCGACACCAAGAAGCAGAGA | qPCR | This study |
|  | CTNNB1-1601R | GGAATGGCACCCTGCTCACGCAAA | qPCR | This study |
| *CCNK* | CCNK-944F | AGCCAGCCCAACAGCCCAAGAAA | qPCR | This study |
|  | CCNK-1101R | TGGAGGCAACGGTGGATGAGTGGT | qPCR | This study |
| *ACTB* | hACTB-556F | ACTGACTACCTCATGAAGAT | qPCR | This study |
|  | hACTB-845R | ATGGAGTTGAAGGTAGTTTC | qPCR | This study |

**Immunoblotting analysis**

On the indicated days following the induction of amiRNAs or UBE2A, and its mutants, DLD1-TetOff cells were washed twice with ice-cold PBS and scraped with 1 mL of ice-cold PBS, transferred to 1.5-mL tubes, and centrifuged at 8,000 rpm (5,800 g; MX-307, TOMY) for 1 min. After aspiration of the supernatants, the pellets were lysed in a buffer containing 50 mM Tris-HCl (pH 8.0), 150 mM NaCl, 5 mM MgCl_2_, 1 mM EDTA (pH 8.0), 0.5% NP-40, 1 mM DTT and, protease and phosphatase inhibitors. After incubating on ice for 15 min, the samples were centrifuged at 15,000 rpm (20,400 g; MX-307, TOMY) for 15 min at 4 °C. The supernatants were transferred to new tubes and their protein concentrations were determined by the Bradford protein assay (500-0001; BioRad). For immunoblot analysis of H2BK120ub, the samples were also sonicated once for 15 seconds before centrifugation. The chemiluminescent signals were detected using a Chemi-Lumi One L (07880; Nacalai tesque) and a ChemiDocTM XRS Plus system (Bio-Rad).

**Immunoprecipitation assay**

A total of 8 × 10^6^ 293T cells (CRL-1573; ATCC) were spread onto a 150 mm dish one day before transfection. The plasmid vectors were transfected using a Polyethylenimine MAX^TM^ (MW 40,000: 24765-100; Polysciences). The amounts of the respective plasmids transfected into 293T cells are listed in the tables below.

Approximately 40 h post-transfection, the cells were washed twice with 10 mL of ice-cold PBS, scraped with 1 mL of ice-cold PBS, and transferred to 1.5-mL tubes. After centrifugation at 8,000 rpm (5,800 g; MX-307, TOMY) for 1 min, the cell pellets were lysed in a buffer containing 50 mM Tris-HCl (pH 8.0), 150 mM NaCl, 5 mM MgCl_2_, 1 mM EDTA (pH 8.0), 0.5% NP-40 and, protease and phosphatase inhibitors. After incubation of the samples on ice for 15 min, they were centrifuged at 15,000 rpm (20,400 g; MX-307, TOMY) for 15 min at 4 °C. The supernatants were transferred to new tubes, and the protein concentrations were determined by the Bradford protein assay. Equal amounts of protein lysates were incubated with anti-DYKDDDDK tag antibody beads (018-22783; FUJIFILM) or ANTI-FLAG^®^ M2 Agarose Affinity Gel (A2220; Sigma Aldrich) for 3 h at 4 °C with rotation. After washing the agarose beads five times with a wash buffer containing 50 mM Tris-HCl (pH 8.0), 150 mM NaCl, 0.1% NP-40, flag-tagged protein complexes were eluted by addition of a 3 × FLAG peptide (F4799; Sigma Aldrich: GEN-3-FLAG; Protein Ark). After the SDS loading buffer was added to the eluted complex, they were incubated for 5 min at 95 °C. The samples were then analyzed via immunoblotting. The chemiluminescent signals were detected using a Chemi-Lumi One L (07880; Nacalai tesque) and a ChemiDoc^TM^ XRS Plus system (Bio-Rad). The signal intensities of the chemiluminescent images were quantified using the Image Lab^TM^ software (Bio-Rad). Experiments were repeated at least twice.

| Fig. 4C |  | | | | | | |
| --- | --- | --- | --- | --- | --- | --- | --- |
|  | **Plasmids (μg)** | | | | | | |
|  | **Lanes 1/2** | **Lane 3** | **Lane 4** | **Lane 5** | **Lane 6** | **Lane 7** | **Lane 8** |
| 1.EGFP | 2 | 5 | 4 | 2.5 | 3.5 | 2.5 | 1 |
| 2.flag-SPT5 | 0 | 1 | 1 | 1 | 1 | 1 | 1 |
| 3.myc-β-Catenin-S33Y | 1.5 | 0 | 0 | 0 | 1.5 | 1.5 | 1.5 |
| 4.myc-CDC73 | 1 | 0 | 1 | 1 | 0 | 1 | 1 |
| 5.myc-CDK9 | 1.5 | 0 | 0 | 1.5 | 0 | 0 | 1.5 |
| 6.myc-RPB2 | 1 | 1 | 1 | 1 | 1 | 1 | 1 |
| 8.myc-PAF1 | 1 | 1 | 1 | 1 | 1 | 1 | 1 |
| 9.myc-TFIIS | 1.5 | 1.5 | 1.5 | 1.5 | 1.5 | 1.5 | 1.5 |
| 10.myc-RPB5 | 0.5 | 0.5 | 0.5 | 0.5 | 0.5 | 0.5 | 0.5 |
| Total (µg) | 10 | 10 | 10 | 10 | 10 | 10 | 10 |

| Fig. 4D |  | | | | | | |
| --- | --- | --- | --- | --- | --- | --- | --- |
|  | **Plasmids (μg)** | | | | | | |
|  | **Lanes 1/2** | **Lane 3** | **Lane 4** | **Lane 5** | **Lane 6** | **Lane 7** | **Lane 8** |
| 1.EGFP | 3.0 | 5.5 | 4.5 | 3 | 4.0 | 3.0 | 1.5 |
| 2.flag-PAF1 | 0 | 1.5 | 1.5 | 1.5 | 1.5 | 1.5 | 1.5 |
| 3.myc-β-Catenin S33Y | 1.5 | 0 | 0 | 0 | 1.5 | 1.5 | 1.5 |
| 4.myc-SPT5 | 1 | 1 | 1 | 1 | 1 | 1 | 1 |
| 5.myc-CDC73 | 1 | 0 | 1 | 1 | 0 | 1 | 1 |
| 6.myc-CDK9 | 1.5 | 0 | 0 | 1.5 | 0 | 0 | 1.5 |
| 7.myc-RPB5 | 0.5 | 0.5 | 0.5 | 0.5 | 0.5 | 0.5 | 0.5 |
| 8.myc-TFIIS | 1.5 | 1.5 | 1.5 | 1.5 | 1.5 | 1.5 | 1.5 |
| Total (µg) | 10 | 10 | 10 | 10 | 10 | 10 | 10 |

| Fig. 4E |  | | | | | | |
| --- | --- | --- | --- | --- | --- | --- | --- |
|  | **Plasmids (μg)** | | | | | | |
|  | **Lanes 1/2** | **Lane 3** | **Lane 4** | **Lane 5** | **Lane 6** | **Lane 7** | **Lane 8** |
| 1.EGFP | 3.0 | 5.5 | 4.5 | 3 | 4 | 3 | 1.5 |
| 2.flag-TFIIS | 0 | 1.5 | 1.5 | 1.5 | 1.5 | 1.5 | 1.5 |
| 3.myc-β-Catenin S33Y | 1.5 | 0 | 0 | 0 | 1.5 | 1.5 | 1.5 |
| 4.myc-SPT5 | 1 | 1 | 1 | 1 | 1 | 1 | 1 |
| 5.myc-CDC73 | 1 | 0 | 1 | 1 | 0 | 1 | 1 |
| 6.myc-CDK9 | 1.5 | 0 | 0 | 1.5 | 0 | 0 | 1.5 |
| 7.myc-PAF1 | 1 | 1 | 1 | 1 | 1 | 1 | 1 |
| 8.myc-RPB3 | 0.5 | 0.5 | 0.5 | 0.5 | 0.5 | 0.5 | 0.5 |
| 9.myc-RPB5 | 0.5 | 0.5 | 0.5 | 0.5 | 0.5 | 0.5 | 0.5 |
| Total (µg) | 10 | 10 | 10 | 10 | 10 | 10 | 10 |

| Fig. S5B |  | | | | |
| --- | --- | --- | --- | --- | --- |
|  | **Plasmids (μg)** | | | | |
|  | **Lanes 1/2** | **Lane 3** | **Lane 4** | **Lane 5** | **Lane 6** |
| 1.EGFP | 2.5 | 5 | 3.5 | 2.5 | 1 |
| 2.flag-SPT5 | 0 | 1.5 | 1.5 | 1.5 | 1.5 |
| 3.myc-PAF1 | 1.5 | 0 | 0 | 1.5 | 1.5 |
| 4.myc-β-Catenin S33Y | 1 | 0 | 0 | 1 | 1 |
| 5.myc-CDC73 | 1.5 | 0 | 1.5 | 0 | 1.5 |
| 6. myc-TFIIS | 1 | 1 | 1 | 1 | 1 |
| 7.myc-RPB5 | 0.5 | 0.5 | 0.5 | 0.5 | 0.5 |
| 8.myc-UBE2A | 2 | 2 | 2 | 2 | 2 |
| Total (µg) | 10 | 10 | 10 | 10 | 10 |

| Fig. S5C |  | | | | | | |
| --- | --- | --- | --- | --- | --- | --- | --- |
|  | **Plasmids (μg)** | | | | | | |
|  | **Lanes 1/2** | **Lane 3** | **Lane 4** | **Lane 5** | **Lane 6** | **Lane 7** | **Lane 8** |
| 1.EGFP | 2 | 5 | 4 | 2.5 | 3.5 | 2.5 | 1 |
| 2.flag-SPT5 | 0 | 1 | 1 | 1 | 1 | 1 | 1 |
| 3.myc-β-Catenin S33Y | 1.5 | 0 | 0 | 0 | 1.5 | 1.5 | 1.5 |
| 4.myc-NELFE | 1 | 0 | 1 | 1 | 0 | 1 | 1 |
| 5.myc-CDK9 | 1.5 | 0 | 0 | 1.5 | 0 | 0 | 1.5 |
| 6.myc-RPB2 | 1 | 1 | 1 | 1 | 1 | 1 | 1 |
| 8.myc-PAF1 | 1 | 1 | 1 | 1 | 1 | 1 | 1 |
| 9.myc-TFIIS | 1.5 | 1.5 | 1.5 | 1.5 | 1.5 | 1.5 | 1.5 |
| 10.myc-RPB5 | 0.5 | 0.5 | 0.5 | 0.5 | 0.5 | 0.5 | 0.5 |
| Total (µg) | 10 | 10 | 10 | 10 | 10 | 10 | 10 |

| Fig. S5D |  | | | | | | |
| --- | --- | --- | --- | --- | --- | --- | --- |
|  | **Plasmids (μg)** | | | | | | |
|  | **Lanes 1/2** | **Lane 3** | **Lane 4** | **Lane 5** | **Lane 6** | **Lane 7** | **Lane 8** |
| 1.EGFP | 3.0 | 5.5 | 4.5 | 3 | 4.0 | 3.0 | 1.5 |
| 2.flag-PAF1 | 0 | 1.5 | 1.5 | 1.5 | 1.5 | 1.5 | 1.5 |
| 3.myc-β-Catenin S33Y | 1.5 | 0 | 0 | 0 | 1.5 | 1.5 | 1.5 |
| 4.myc-CDC73 | 1 | 0 | 1 | 1 | 0 | 1 | 1 |
| 5.myc-CDK9 | 1.5 | 0 | 0 | 1.5 | 0 | 0 | 1.5 |
| 6.myc-RPB2 | 1 | 1 | 1 | 1 | 1 | 1 | 1 |
| 7.myc-RPB5 | 0.5 | 0.5 | 0.5 | 0.5 | 0.5 | 0.5 | 0.5 |
| 8.myc-TFIIS | 1.5 | 1.5 | 1.5 | 1.5 | 1.5 | 1.5 | 1.5 |
| Total (µg) | 10 | 10 | 10 | 10 | 10 | 10 | 10 |

| Fig. S5E |  | | | | | | |
| --- | --- | --- | --- | --- | --- | --- | --- |
|  | **Plasmids (μg)** | | | | | | |
|  | **Lanes 1/2** | **Lane 3** | **Lane 4** | **Lane 5** | **Lane 6** | **Lane 7** | **Lane 8** |
| 1.EGFP | 3.0 | 5.5 | 4.5 | 3 | 4.0 | 3.0 | 1.5 |
| 2.flag-PAF1 | 0 | 1.5 | 1.5 | 1.5 | 1.5 | 1.5 | 1.5 |
| 3.myc-β-Catenin S33Y | 1.5 | 0 | 0 | 0 | 1.5 | 1.5 | 1.5 |
| 4.myc-SPT5 | 1 | 1 | 1 | 1 | 1 | 1 | 1 |
| 5.myc-NELFE | 1 | 0 | 1 | 1 | 0 | 1 | 1 |
| 6.myc-CDK9 | 1.5 | 0 | 0 | 1.5 | 0 | 0 | 1.5 |
| 7.myc-RPB5 | 0.5 | 0.5 | 0.5 | 0.5 | 0.5 | 0.5 | 0.5 |
| 8.myc-TFIIS | 1.5 | 1.5 | 1.5 | 1.5 | 1.5 | 1.5 | 1.5 |
| Total (µg) | 10 | 10 | 10 | 10 | 10 | 10 | 10 |

| Fig. S5F |  | | | | | | |
| --- | --- | --- | --- | --- | --- | --- | --- |
|  | **Plasmids (μg)** | | | | | | |
|  | **Lanes 1/2** | **Lane 3** | **Lane 4** | **Lane 5** | **Lane 6** | **Lane 7** | **Lane 8** |
| 1.EGFP | 3.0 | 5.5 | 4.5 | 3 | 4 | 3 | 1.5 |
| 2.flag-TFIIS | 0 | 1.5 | 1.5 | 1.5 | 1.5 | 1.5 | 1.5 |
| 3.myc-β-Catenin S33Y | 1.5 | 0 | 0 | 0 | 1.5 | 1.5 | 1.5 |
| 4.myc-RPB2 | 1 | 1 | 1 | 1 | 1 | 1 | 1 |
| 5.myc-NELFE | 1 | 0 | 1 | 1 | 0 | 1 | 1 |
| 6.myc-CDK9 | 1.5 | 0 | 0 | 1.5 | 0 | 0 | 1.5 |
| 7.myc-PAF1 | 1 | 1 | 1 | 1 | 1 | 1 | 1 |
| 8.myc-RPB3 | 0.5 | 0.5 | 0.5 | 0.5 | 0.5 | 0.5 | 0.5 |
| 9.myc-RPB5 | 0.5 | 0.5 | 0.5 | 0.5 | 0.5 | 0.5 | 0.5 |
| Total (µg) | 10 | 10 | 10 | 10 | 10 | 10 | 10 |

| Fig. 5C |  | | | | | | |
| --- | --- | --- | --- | --- | --- | --- | --- |
|  | **Plasmids (μg)** | | | | | | |
|  | **Lanes 1/2** | **Lane 3** | **Lane 4** | **Lane 5** | **Lane 6** | **Lane 7** | **Lane 8** |
| 1.EGFP | 3.0 | 5.5 | 4.5 | 3 | 4 | 3 | 1.5 |
| 2.flag-NELFA | 0 | 1.5 | 1.5 | 1.5 | 1.5 | 1.5 | 1.5 |
| 3.myc-β-Catenin S33Y | 1.5 | 0 | 0 | 0 | 1.5 | 1.5 | 1.5 |
| 4.myc-SPT5 | 1 | 1 | 1 | 1 | 1 | 1 | 1 |
| 5.myc-CDC73 | 1 | 0 | 1 | 1 | 0 | 1 | 1 |
| 6.myc-CDK9 | 1.5 | 0 | 0 | 1.5 | 0 | 0 | 1.5 |
| 7.myc-PAF1 | 1 | 1 | 1 | 1 | 1 | 1 | 1 |
| 8.myc-RPB3 | 0.5 | 0.5 | 0.5 | 0.5 | 0.5 | 0.5 | 0.5 |
| 9.myc-RPB5 | 0.5 | 0.5 | 0.5 | 0.5 | 0.5 | 0.5 | 0.5 |
| Total (µg) | 10 | 10 | 10 | 10 | 10 | 10 | 10 |

| Fig. S6C |  | | | | | | |
| --- | --- | --- | --- | --- | --- | --- | --- |
|  | **Plasmids (μg)** | | | | | | |
|  | **Lanes 1/2** | **Lane 3** | **Lane 4** | **Lane 5** | **Lane 6** | **Lane 7** | **Lane 8** |
| 1.EGFP | 3.0 | 5.5 | 4.5 | 3 | 4 | 3 | 1.5 |
| 2.flag-NELFA | 0 | 1.5 | 1.5 | 1.5 | 1.5 | 1.5 | 1.5 |
| 3.myc-β-Catenin S33Y | 1.5 | 0 | 0 | 0 | 1.5 | 1.5 | 1.5 |
| 4.myc-SPT5 | 1 | 1 | 1 | 1 | 1 | 1 | 1 |
| 5.myc-NELFE | 1 | 0 | 1 | 1 | 0 | 1 | 1 |
| 6.myc-CDK9 | 1.5 | 0 | 0 | 1.5 | 0 | 0 | 1.5 |
| 7.myc-PAF1 | 1 | 1 | 1 | 1 | 1 | 1 | 1 |
| 8.myc-RPB3 | 0.5 | 0.5 | 0.5 | 0.5 | 0.5 | 0.5 | 0.5 |
| 9.myc-RPB5 | 0.5 | 0.5 | 0.5 | 0.5 | 0.5 | 0.5 | 0.5 |
| Total (µg) | 10 | 10 | 10 | 10 | 10 | 10 | 10 |

**Drugs**

THZ531 (S6595; Selleck), dinaciclib (S2768; Selleck), and 5-Fluorouracil (064-01403; FUJIFILM) were dissolved in dimethyl sulfoxide (DMSO; 043-07216; FUJIFILM).

**Cell-proliferation assay of TetOff cells**

A total of 2 × 10^4^ DLD1-TetOff cells or 3 × 10^4^ LS174T-TetOff cells were seeded in the each well of 12-well plates on day 2 following induction of amiRNAs. The cells from three independent wells were collected and their numbers were determined using a BECKMAN COULTER Z series particle counter (BECKMAN COULTER), and compared with those of cells not expressing the amiRNAs.

**Cell-proliferation assay of HT29 cells**

A total of 5 × 10^4^ HT29 cells (HTB-38; ATCC) pretreated with CDK inhibitors or 5-FU were seeded in the each well of 12-well plates. The cells from three independent wells were collected and their numbers were determined using a BECKMAN COULTER Z series particle counter (BECKMAN COULTER), and compared with those of the DMSO-pretreated cells.

To analyze the effects of CDK inhibitors or 5-FU on the proliferation of HT29 cells, they were cultured in the presence of the drugs and their numbers were determined as described above, and compared with those of the DMSO-treated cells.

**Subcutaneous transplantation of LS174T-TetOff and HT29 cells**

For the analysis of *in vivo* tumor growth, 2 × 10^6^ LS174T-TetOff cells (Fig. 2) or 1 × 10^6^ HT29 cells (Fig. 7) were subcutaneously injected into the male NOG mouse (CIEA) at 12 weeks old with 50 μL of BD Matrigel Matrix (354234; ThermoFisher Scientific). For the LS174T-TetOff-amiR-*PAF1*_1 cells, six transplanted tumors per group were analyzed. For the LS174T-TetOff-amiR-*CTNNB1*_1 cells, four transplanted tumors per group were analyzed. For the analysis of HT29 cells pretreated with CDK inhibitors or 5FU, four to six transplanted tumors per group were analyzed.

For the analysis of *PAF1* in LS174T-TetOff cells, expression of an amiRNA targeting *PAF1* was induced at the same time as transplantation or induced 3 days before transplantation as illustrated in Fig. 2. For the analysis of *CTNNB1* in the LS174T-TetOff cells, expression of an amiRNA targeting *CTNNB1* was induced at the same time as transplantation. For the analysis of the tumors without the amiRNAs expression, the tumor-bearing mice were administered 5% sucrose drinking water containing 0.1 mg/mL DOX. To analyze the tumors with the amiRNAs expression, the tumor-bearing mice were administered 5% sucrose drinking water without DOX. The drinking water was replaced with fresh water every three to four days (twice a week) for both groups.

The tumor diameters were determined using a caliper at the indicated days in Figs 2 and 7. Tumor volumes were calculated using the formula V = W^2^ × L × 2, where V, W, and L are the volume, width, and length of the tumor, respectively. After the tumors were excised at the end of the transplantation assays, their weights were determined, and they were fixed with 10% formalin in PBS for one or two days. At the same time, RNA was purified from the excised tumors using TRI Reagent^®^ for the qPCR analysis of *LGR5* expression. The animal experiments were approved by the Animal Care and Use Committee of FUKUI University (R04023).

**Histological analysis**

After fixation of the tumor tissues with 10% formalin, the formalin-fixed, paraffin-embedded sections were stained with hematoxylin and eosin (H&E, 30082 and 32043; Muto pure chemicals) according to the standard method. Immuno-hisotochemistry was performed as previously described [1] using an anti-Cytokeratin 20 antibody (ab76126; Abcam; RRID:AB_1310117). Optical microscope images were taken using the BX63 microscope (Olympus Life Science) with the DP73 microscope color camera (Olympus Life Science) or the Qimaging RETIGA EXi digital camera (Roper Scientific).

**Alcian blue stain (pH 2.5)**

After the formalin-fixation, paraffin-embedded sections were deparafinized and hydrated, they were incubated in acetic acid and stained with pH2.5 Alcian Blue 8GX (CDX-A0001; AdipoGen^®^) for 30 min. The sections were further stained with a nuclear fast red (N0184; TGI) for 5 min.

**Construction of luciferase reporter plasmids**

The upstream and downstream DNA regions of the *LGR5* transcription start site (TSS) were amplified via PCR using the DNA isolated from DLD1 cells using a PrimeSTAR^®^ GXL DNA Polymerase (R050A; TAKARA). After the amplified fragments were cloned into a pBS plasmid, the cloned DNA regions were inserted into the pGL4.10 [luc2] plasmid vector (E6651; Promega). The DNA regions upstream of the TSS of *LGR5* were inserted into a multi-cloning site of pGL4.10 [luc2] vector, whereas the downstream regions were inserted into an XbaI site between *luc2* and bpA. The oligonucleotide primer sequences used for cloning the *LGR5* gene are listed below.

| **Oligo nucleotide primers used for construction of *LGR5* *luciferase* reporter plasmids** | | |
| --- | --- | --- |
| ***LGR5* positions to be cloned** | **Primer names** | **Sequence** |
| (-)6000 - (-)1 | LGR5-6000F | ATATGGATCCGCTAGCCCTATCAAAATACCACATCATTTTTCAA |
| (-)5038 - (-)1 | LGR5-5038F | ATATGGATCCGCTAGCGGACTGGTATCCAGAATCTACAAGAAAC |
| (-)3999 - (-)1 | LGR5-3999F | ATATGGATCCGCTAGCGGGTGTATGCCCCATCCAAGTCCAAAAC |
| (-)3037 - (-)1 | LGR5-3037F | ATATGGATCCGCTAGCCAGGGGCACAATGCCTAGAACCTCTTTG |
| (-)1998 - (-)1 | LGR5-1998F | ATATGGATCCGCTAGCGTAGGAGAAGGGTGTGGGATTAAAAACT |
| (-)1015 - (-)1 | LGR5-1015F | ATATGGATCCGCTAGCGCATCGATTTACTAAAAGTGACTTCTAG |
| Promoter (reverse primer) | hLGR5-1R | ATGCATGTCGACCTCGAGGGTGCCCGAAGTAGGGGGCCACGAGCCGGAC |
| 1st intron (forward primer) | hLGR5-213F | ATGCGTCGACTCTAGAGTAAGTACTTCCCCACGTCACTCCGGGA |
| (+)213 - (+)905 | hLGR5-905R | ATATGTCGACTCTAGAGCAGCCAAAGGTAAAGATAACGTTCTGG |
| (+)213 - (+)1494 | hLGR5-1494R | ATATGTCGACTCTAGAGCAAGCCTCTTCATGTTTCTTCCCTCCA |
| (+)213 - (+)2023 | hLGR5-2023R | ATATGTCGACTCTAGAGTCTGGGGGTACTGCACACCTCCCTTGC |
| (+)213 - (+)2972 | hLGR5-2972R | ATATGTCGACTCTAGATAGTAGTTCTGGCTTTTTACCACATC |
| (+)213 - (+)4009 | hLGR5-4009R | ATATGTCGACTCTAGACACATTTATGATATTAAGGCGGTGCCTG |

**Luciferase reporter assay**

293T cells on a 24-well plate were transfected with 0.025 μg of the pGL4.10 [luc2] or pGL4.10-h*LGR5* reporter plasmid vectors, 0.025 μg of pGL4.75 [hRuc/CMV] plasmid vector (E6931; Promega), and 0.125 μg of the respective plasmid vectors expressing EGFP, β-catenin-S33Y, PAF1C components or its related molecules, or NELF components, using a PEI MAXTM. Four wells of a 24-well plate were transfected with a same plasmid set in quadruplicate. The total amount of plasmids transfected into 293T cells in a well were adjusted to the same with the plasmid vector expressing EGFP. The luciferase activities were determined using a Dual-Luciferase Reporter Assay System (E1910; Promega) at ~40 h post-transfection. The experiments were repeated at least twice. The pGL4.75 [hRuc/CMV] plasmid vector (E693A; Promega) was used as a reference for the transfection efficiency. The luciferin signals were detected using a GloMax-Multi+ detection system (Promega).

To analyze the effects of the drugs on the *LGR5 luc* reporter activity, 293T cells were treated with THZ531, dinaciclib, or 5-FU at the indicated concentrations for 24 h after the transfection (Fig. 7). Then, cells were harvested and the luciferin signals were detected using a GloMax-Multi+ detection system (Promega).

To analyze the β-catenin-mediated transcriptional activity of the HT29 cells that were derived from *in vivo* tumors, TOPflash and FOPflash reporters were used as described previously [3].

**Statistical analysis**

Statistical analyses were carried out using the Student’s t-test, and statistical significance was set at *P*-values < 0.05. Calculations, graphs and statistics were generated using Microsoft Excel.

**Supplementary References**

1. Aoki K, Kakizaki F, Sakashita H, Manabe T, Aoki M, Taketo MM. Suppression of colonic polyposis by homeoprotein CDX2 through its nontranscriptional function that stabilizes p27^Kip1^. Cancer Res. 2011;71: 593-602.

2. Myburgh R, Cherpin O, Schlaepfer E, Rehrauer H, Speck RF, Krause, KH. Optimization of Critical Hairpin Features Allows miRNA-based Gene Knockdown Upon Single-copy Transduction. Mol. Ther. Nucleic Acids. 2014;3:e207.

3. Aoki K, Aoki M, Sugai M, Harada N, Miyoshi H, Tsukamoto T, et al. Chromosomal instability by β-catenin/TCF transcription in APC or β-catenin mutant cells. Oncogene 2007;26:3511-3520.

4. Konig H, Moll J, Ponta H, Herrlich P. Trans-acting factors regulate the expression of CD44 splice variants. EMBO J. 1996;15:4030-4039.

5. Liu L, Yan C, Tao S. Association of MUC2, MUC5AC and MUC5B genes with the recurrence of nasal polyps. Exp. Ther. Med. 2020;20:1808-1814.

**Supplementary Fig. 1**

**Supplementary Fig. 1: TetOff cells that expressed amiRNAs targeting *PAF1* and *CTNNB1*.**

**A** Line graphs of the qPCR data showing the relative expression levels (mean ± SD) of *PAF1*, *CTNNB1*, and *SPT5* upon expression of the respective amiRNAs targeting *PAF1* (orange) or *CTNNB1* (blue) for the indicated days in DLD1-TetOff cells (left) and LS174T-TetOff (right) cells, compared with those in cells not expressing the amiRNAs (grey). RNA was purified from the TetOff cells at the indicated days following expression of the amiRNAs, and the qPCR assay was performed. The TetOff cells that expressed an amiRNA against *EGFP* was used as the control (black).

**B** Line graphs showing the number of DLD1-TetOff (top) and LS174T-TetOff (bottom) cells upon expression of the respective amiRNAs targeting *PAF1* (orange) or *CTNNB1* (blue) for the indicated days, compared with that of cells not expressing the amiRNAs (grey). After the TetOff cells were spread onto 12-well plates on day 2 following expression of the amiRNAs, their cell number was determined at the indicated days using the Beckman coulter counter.

**C** Immunoblot images showing the protein expression of β-catenin and ubH2B upon expression of the respective amiRNAs targeting *PAF1* or *CTNNB1* in DLD1-TetOff cells for the indicated days, compared with that of cells not expressing the amiRNAs. The TetOff cells that expressed the amiRNA against *EGFP* was used as the control. β-Actin was used as the loading control. The cells were harvested on day 3 and 7 following expression of the amiRNAs, and the protein expression levels of β-catenin and ubH2B was analyzed by the western blotting analysis.

**Supplementary Fig. 2**

**Supplementary Fig. 2: Control of colon cancer stemness by PAF1.**

**A** Line graphs of the qPCR data showing the relative expression levels (mean ± SD) of cancer stemness-related genes, and *SPDEF* and *MUC* genes upon expression of the respective amiRNAs targeting *PAF1* (orange) or *CTNNB1* (blue) for the indicate days in DLD1-TetOff (top) and LS174T-TetOff (bottom) cells, compared with those in cells not expressing the amiRNAs (grey). RNA was purified from the TetOff cells at the indicated days following expression of the amiRNAs, and the qPCR assay was performed.

**B** Line graphs of the qPCR data showing the relative expression levels (mean ± SD) of cancer stemness-related genes upon expression of amiRNA-*CTNNB1*_2 (top) or amiRNA-*CTNNB1*_3 (bottom) for the indicated days in DLD1-TetOff cells, compared with those in cells not expressing the amiRNAs (grey). RNA was purified from the TetOff cells at the indicated days following expression of the amiRNAs, and the qPCR assay was performed.

**Supplementary Fig. 3**

**Supplementary Fig. 3: TetOff cells that expressed amiRNAs targeting *CDC73* and *CTR9*.**

**A** Line graphs showing the number of DLD1-TetOff cells upon expression of amiR-*CDC73* (left) or amiR-*CTR9* (right) for the indicated days, compared with that of cells not expressing the amiRNAs. After the TetOff cells were spread onto 12-well plates on day 2 following expression of the amiRNAs, their cell number was determined at the indicated days using the Beckman coulter counter.

**B** Line graphs of the qPCR data showing the relative expression levels (mean ± SD) of cancer stemness-related or differentiation marker genes upon expression of amiR-*CDC73* (top) or amiR-*CTR9* (bottom) for the indicated days in DLD1-TetOff cells, compared with those in cells not expressing the amiRNAs. RNA was purified from the TetOff cells at the indicated days following expression of the amiRNAs, and the qPCR assay was performed.

**Supplementary Fig. 4**

**Supplementary Fig. 4: TetOff cells that expressed UBE2A mutants or an amiRNA targeting *CCNK*.**

**A** Immunoblot images showing the expression of flag-tagged UBE2A mutants and endogenous ubH2B upon inducible expression of UBE2A-C88S or UBE2A-C88V in DLD1-TetOff cells for 2 days, compared with that in cells not expressing the UBE2A mutants. Ubiquitylated UBE2A-C88S migrated slower than non-ubiquitylated UBE2A because of binding to ubiquitin (Ub).

**B** Line graphs showing the number of DLD1-TetOff cells upon expression of UBE2A-C88S (left) or UBE2A-C88V (right) for the indicated days, compared with that of cells not expressing the UBE2A mutants. After the TetOff cells were spread onto 12-well plates on day 2 following expression of the UBE2A mutants, their cell number was determined at the indicated days using the Beckman coulter counter.

**C** Line graphs of the qPCR data showing the relative expression levels (mean ± SD) of cancer stemness-related or differentiation marker genes upon expression of of UBE2A-C88S (top) or UBE2A-C88V (bottom) in DLD1-TetOff cells for the indicated days in DLD1-TetOff cells, compared with those in cells not expressing the UBE2A mutants. RNA was purified from the TetOff cells at the indicated days following expression of the UBE2A mutants, and the qPCR assay was performed.

**D** Line graph showing the number of DLD1-TetOff cells upon expression of amiR-*CCNK* for the indicated days, compared with that of cells not expressing the amiRNA. After the TetOff cells were spread onto 12-well plates on day 2 following expression of an amiRNA against *CCNK*, their cell number was determined at the indicated days using the Beckman coulter counter.

**E** Line graphs of the qPCR data showing the relative expression levels (mean ± SD) of cancer stemness-related or differentiation marker genes upon expression of amiR-*CCNK* in DLD1-TetOff cells for the indicated days, compared with those in cells not expressing the amiRNA. RNA was purified from the TetOff cells at the indicated days following expression of the amiRNA, and the qPCR assay was performed.

**Supplementary Fig. 5**

**Supplementary Fig. 5: Enhanced formation of Pol II-DSIF-PAF1C complex by stable β-catenin.**

**A** Immunoblot images showing expression of the myc-tagged molecules used in the immunoprecipitation (IP) assays in this study. On day 2 after transfection of the plasmid vectors expressing the indicated proteins into 293T cells, the cells were harvested and the western blotting assay was performed.

**B** Immunoblot images of the IP assay showing the effects of β-catenin-S33Y and CDC73 on the complex formation of SPT5 containing TFIIS and UBE2A. The cells were harvested on day 2 after transfection of the plasmid vectors expressing the indicated proteins into 293T cells, and IP was performed. After IP of flag-tagged SPT5, the amounts of the co-immunoprecipitated myc-tagged molecules were analyzed via immunoblotting. The cell lysates used for the IP assay are indicated at the bottom (input). Longer exposure gel images were also shown. Purple asterisks indicate signals of the light chain of immunoglobulin derived from anti-flag antibody-conjugated agarose beads used for the IP assay. Orange arrows indicate weak signals of myc-UBE2A.
**C** Immunoblot images of the IP assay showing the effects of β-catenin-S33Y, NELFE, and CDK9 on the complex formation of SPT5 containing TFIIS. The cells were harvested on day 2 after transfection of the plasmid vectors expressing the indicated proteins into 293T cells, and IP was performed. After IP of flag-tagged SPT5, the amounts of the co-immunoprecipitated myc-tagged molecules were analyzed via immunoblotting. The cell lysates used for the IP assay are indicated at the bottom (input).

**D**, **E** Immunoblot images of the IP assays showing the effects of β-catenin-S33Y, CDC73 (**D**), NELFE (**E**), and CDK9 on the complex formation of PAF1C containing TFIIS. The cells were harvested on day 2 after transfection of the plasmid vectors that expressed the indicated proteins into 293T cells, and IP was performed. After IP of flag-tagged PAF1, the amounts of the co-immunoprecipitated myc-tagged molecules were analyzed via immunoblotting. In (**D**), myc-RPB2 was expressed whereas myc-SPT5 was expressed in (**E**). The cell lysates used for the IP assay are indicated at the bottom (input). A longer exposure gel image was also shown.

**F** Immunoblot images of the IP assay showing the effects of β-catenin-S33Y, NELFE, and CDK9 on the complex formation of TFIIS containing RPB2. The cells were harvested on day 2 after transfection of the plasmid vectors that expressed the indicated proteins into 293T cells, and IP was performed. After IP of flag-TFIIS, the amounts of the co-immunoprecipitated myc-tagged molecules were analyzed via immunoblotting. Cell lysates used for the IP assay are indicated at the bottom (input).

**Supplementary Fig. 6**

**Supplementary Fig. 6: Contribution of NELF to *LGR5* expression cooperatively with stable β-catenin.**

**A** Line graph showing the number of DLD1-TetOff cells upon expression of both amiR-*NELFA* and amiR-*NELFE* (blue) for the indicated days, compared with that of cells not expressing the amiRNAs (grey). The TetOff cells were spread onto 12-well plates on day 2 following expression of the amiRNAs against *NELFA* and *NELFE,* and their cell number was determined at the indicated days using the Beckman coulter counter.

**B** Line graphs of the qPCR data showing the relative expression levels (mean ± SD) of cancer stemness-related or differentiation marker genes upon expression of both amiR-*NELFA* and amiR-*NELFE* (blue) in DLD1-TetOff cells for the indicated days, compared with those in cells not expressing the amiRNAs (grey). RNA was purified from the TetOff cells at the indicated days following expression of the amiRNAs, and the qPCR assay was performed.

**C** Immunoblot images of the IP assays showing the effect of β-catenin-S33Y on the complex formation of flag-NELFA containing SPT5, PAF1, and RPBs. The cells were harvested on day 2 after transfection of the plasmid vectors that expressed the indicated proteins into 293T cells, and IP was performed. After IP of flag-tagged NELFA, the amounts of the co-immunoprecipitad myc-tagged molecules were analyzed via immunoblotting. The cell lysates used for the IP are indicated at the bottom (input).

**Supplementary Fig. 7**

**Supplementary Fig. 7: Reduced expression levels of *ID1* and *CD44v9* by CDK12 inhibitors.**

**A** Bar graphs of the qPCR data showing the relative expression levels (mean ± SD) of *ID1* and *CD44v9* upon treatment of the human colon cancer cells HT29 (top), SW620 (middle), and SW837 (bottom) with THZ531 (pink), dinaciclib (blue), or 5-FU (yellow) at the indicated concentrations for 24 h, compared with those in the DMSO-treated cells (grey). RNA was purified after treatment of the cells with the indicated drugs for 24 h, and the qPCR assay was performed.

**B** Line graphs showing the number of HT29 cells at the indicated days after the treatment with 200 nM of THZ531 (top), 20 nM of dinaciclib (middle), and 5 μM of 5-FU (bottom), compared with that of the DMSO-treated cells (grey). The indicated drugs were added to the culture media one day after the HT29 cells were spread onto 12-well plates, and their cell number was determined at the indicated days after the treatment using the Beckman coulter counter. Culture media were replaced every day with fresh ones containing the drugs.

**Supplementary Fig. 8**

**Supplementary Fig. 8: Suppression of colon cancer stemness by CDK12 inhibitors.**

**A**-**C** Optical micrographs showing the H&E-stained subcutaneously-transplanted HT29 cells pretreated with DMSO (**A**), THZ531 (**B**), and dinaciclib (**C**). Scale bars, 200 μm.

**D** Optical micrographs showing the alcian blue (pH 2.5)-stained sections of a subcutaneously transplanted THZ531-tumor. A bottom photo is the magnified image of top one. Scale bars, 200 μm (top) and 100 μm (bottom).

**E** Bar graph of the qPCR data showing the relative expression level (mean ± SD) of *LGR5* in the *in vivo* tumors originated from the cells pretreated with THZ531 (pink) or dinaciclib (blue), compared with that in tumors originated from DMSO-pretreated cells (grey).

**F** Bar graph showing the luciferase activity (mean ± SD) of the TOPflash reporter relative to that of FOPflash in the cultured cells derived from THZ531- (pink) or dinaciclib-tumors (blue), compared with that in cells derived from DMSO-pretreated cells (grey).
